# RNase H is an exo- and endoribonuclease with asymmetric directionality, depending on the binding mode to the structural variants of RNA:DNA hybrids

**DOI:** 10.1093/nar/gkab1064

**Published:** 2021-11-12

**Authors:** Hyunjee Lee, HyeokJin Cho, Jooyoung Kim, Sua Lee, Jungmin Yoo, Daeho Park, Gwangrog Lee

**Affiliations:** School of Life Sciences, Gwangju Institute of Science and Technology, Gwangju 61005, Korea; Single-Molecule Biology Laboratory, Gwangju Institute of Science and Technology, Gwangju 61005, Korea; Cell Mechanobiology Laboratory, Gwangju Institute of Science and Technology, Gwangju 61005, Korea; School of Life Sciences, Gwangju Institute of Science and Technology, Gwangju 61005, Korea; Single-Molecule Biology Laboratory, Gwangju Institute of Science and Technology, Gwangju 61005, Korea; Cell Mechanobiology Laboratory, Gwangju Institute of Science and Technology, Gwangju 61005, Korea; School of Life Sciences, Gwangju Institute of Science and Technology, Gwangju 61005, Korea; School of Life Sciences, Gwangju Institute of Science and Technology, Gwangju 61005, Korea; School of Life Sciences, Gwangju Institute of Science and Technology, Gwangju 61005, Korea; Single-Molecule Biology Laboratory, Gwangju Institute of Science and Technology, Gwangju 61005, Korea; Cell Mechanobiology Laboratory, Gwangju Institute of Science and Technology, Gwangju 61005, Korea; School of Life Sciences, Gwangju Institute of Science and Technology, Gwangju 61005, Korea; Cell Mechanobiology Laboratory, Gwangju Institute of Science and Technology, Gwangju 61005, Korea; School of Life Sciences, Gwangju Institute of Science and Technology, Gwangju 61005, Korea; Single-Molecule Biology Laboratory, Gwangju Institute of Science and Technology, Gwangju 61005, Korea; Cell Mechanobiology Laboratory, Gwangju Institute of Science and Technology, Gwangju 61005, Korea

## Abstract

RNase H is involved in fundamental cellular processes and is responsible for removing the short stretch of RNA from Okazaki fragments and the long stretch of RNA from R-loops. Defects in RNase H lead to embryo lethality in mice and Aicardi-Goutieres syndrome in humans, suggesting the importance of RNase H. To date, RNase H is known to be a non-sequence-specific endonuclease, but it is not known whether it performs other functions on the structural variants of RNA:DNA hybrids. Here, we used *Escherichia coli* RNase H as a model, and examined its catalytic mechanism and its substrate recognition modes, using single-molecule FRET. We discovered that RNase H acts as a processive exoribonuclease on the 3′ DNA overhang side but as a distributive non-sequence-specific endonuclease on the 5′ DNA overhang side of RNA:DNA hybrids or on blunt-ended hybrids. The high affinity of previously unidentified double-stranded (ds) and single-stranded (ss) DNA junctions flanking RNA:DNA hybrids may help RNase H find the hybrid substrates in long genomic DNA. Our study provides new insights into the multifunctionality of RNase H, elucidating unprecedented roles of junctions and ssDNA overhang on RNA:DNA hybrids.

## Graphical Abstract

**Graphical Abstract:**
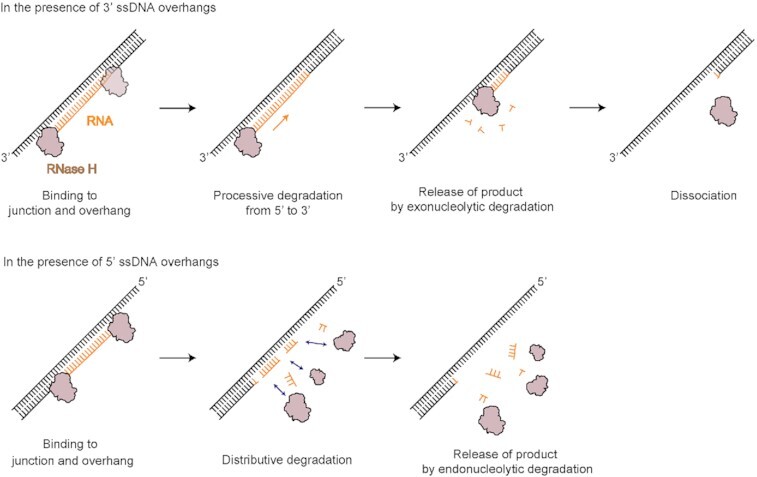
The type of overhang of RNA:DNA hybrids determines asymmetric enzymatic activity.

## INTRODUCTION

RNase H participates in many fundamental genetic processes, such as replication and transcription (1,2). During cellular processes, RNA and DNA heteroduplexes are temporally generated in nucleic acid metabolism via the incorporation of ribonucleotides into an ssDNA strand and the hybridization of RNA and DNA strands (3,4) to form RNA:DNA (termed RNA:DNA hybrid, where ‘:’ indicates hybridization). For genomic stability, cells in all domains of life have utilized RNase H as a simple solution to resolve the issue of RNA:DNA hybrids (5). In bacteria, RNase H participates in the processing of RNA primers from Okazaki fragments during DNA replication and rescues intermittent transcriptional arrest by removing RNA strands from R-loops (6–8). RNase H knockout is lethal in mice due to the lack of mitochondrial DNA synthesis (9), and mutations of RNase H2 lead to Aicardi-Goutières syndrome in humans (10,11).

Over the past decades, many crystal structures of RNase H have been determined, from sources ranging across T4 bacteriophages (12) to bacteria (13,14) and eukaryotes (15,16), revealing the mechanisms of substrate recognition and nucleotidyl transfer. Among the two types of RNase H (i.e. HI/HII in bacteria and H1/H2 in eukaryotes) (17), RNase HI/H1 is found across many species, from viruses and bacteria to animals. Thus, RNase HI/H1 has been intensively studied as a model of two-metal-ion catalysis (18,19), which is key to the cleavage reaction broadly used by nucleases, polymerases, and topoisomerases in various steps of nucleic acid metabolism. To date, RNase HI is widely accepted as a non-sequence-specific endonuclease (16,17) that selectively cleaves the RNA strand in an RNA:DNA hybrid.

Crystal structures of *Bacillus halodurans* RNase HI were solved in complex with an RNA:DNA hybrid (14,20). The binding of RNase H to the hybrid results in direct contact with five consecutive 2′-OH groups on the RNA strand and two phosphates on the DNA strand (14). The active site of RNase H lies in the groove that harbors the RNA and DNA strands. Four ribonucleotides, two on each side of the scissile bond, are required for RNA cleavage at the active site composed of acidic residues (D10, E48 and D70 for *Escherichia coli*) (21) whereas recognition of the DNA backbone is achieved by a phosphate binding pocket at the N-terminus of helix A (T43, N45 and T100 for *E. coli*). RNase HI in *E. coli* is a homolog of RNase H1 in the mitochondria and the nucleus of eukaryotes. In particular, *E. coli* RNase HI is structurally very similar to the C-terminal catalytic domain (RNase HC) of human RNase H1 except for the N-terminal hybrid-binding domain (HBD) (22). From here on, we will refer to both RNase HI and H1 as RNase H.

Nucleic acid enzymes typically recognize the structural features of their substrates, utilize the chemical properties of nucleic acids, and perform organized reactions, i.e. a series of cleavage reactions using directional translocation along polarized nucleic acids (termed enzymatic directionality). For this reason, interaction and catalysis by RNase H should also be influenced by the basic structural features of substrates, such as the 3′ and 5′ ssDNA overhangs of R-loops, different duplex junctions, and the chimeric junction of an RNA–DNA strand (where ‘–’ represents the chimeric covalent linkage between ssRNA and ssDNA) in the genomic DNA. To date, however, structural and biochemical studies on RNase H have focused mainly on simple RNA:DNA hybrids, not on hybrids containing an ssDNA overhang, different duplex junctions or chimeric junctions of the RNA–DNA strand (23).

Many intriguing questions arise as to how RNase H recognizes and responds to structural distortion since chimeric RNA–DNA:DNA hybrids become gradually distorted from the A form of RNA:DNA to the B form of DNA:DNA. Like some enzymes that utilize the hydrolysis of phosphate groups to move in one direction along polarized nucleic acid strands (24,25), does RNase H have specific directionality that relies on features of structural variants of RNA:DNA hybrids during RNA removal? Do these structural features direct the enzymatic activity and recognition of RNase H? Moreover, the dynamic view of RNase H activity has not been examined on structural variants of RNA:DNA hybrids containing physical distortions, RNA–DNA junctions or ssDNA overhangs. The activity of RNase H without HBD has been classified only as an endoribonuclease with nondirectional and distributive modes of action (26,27).

Recent progress in single-molecule (sm) methods has allowed us to explore physiologically relevant dynamic activities such as heterogeneity of activity (28–30), the anatomy of multistep enzyme reactions (31), the relationship between conformational transition and functional change (32–35), hidden complexity (36,37), and metal-ion dynamics (24). In this study, we monitored the enzymatic motion of RNase H using smFRET with high spatiotemporal resolution and investigated the following questions: (i) how RNase H recognizes prominent features in RNA:DNA hybrids; (ii) what structural cues determine the degradation kinetics of RNase H and (iii) how the 3′ or 5′ polarity of the ssDNA overhang of an RNA:DNA hybrid selectively dictates processive or distributive degradation of the enzyme, respectively. We found unprecedented functional properties in which RNase H acts not only as a random and non-sequence-specific distributive endonuclease but also as a 5′-to-3′ directional processive exonuclease, depending on the polarity of the overhangs protruding from RNA:DNA hybrids. Our study provides new insight into the distinct roles of DNA overhangs in the removal of RNA from R-loops and RNA primers from Okazaki fragments.

## MATERIALS AND METHODS

### Protein purification

The *E. coli RNase H gene* was inserted into the pET28a vector, which contains a 6-histidine tag. The cloned vector was confirmed by DNA sequencing, and RNase H was expressed in *E. coli* cells. The fusion plasmid construct was transformed into BL21-Star *E. coli* and expressed in 1 L of LB. Bacterial cultures were grown to an OD_600_ of 0.5. Then, IPTG was added at a final concentration of 1 mM. After shaking for 14–16 hours at 18°C, bacteria were harvested in a rotor at 5000 × *g*, resuspended in 40 ml of buffer (20 mM Tris–HCl (pH 7.5), 500 mM NaCl) and lysed by sonication. The cell lysate was centrifuged for 30 min at 35 000 × *g*. After the supernatant was filtered, the sample was loaded into a HisTrap FF column (5 ml) (GE Healthcare). The protein was eluted through a gradual increase in imidazole (30, 100, 300, 500 mM), and purified RNase H was stored in buffer containing 50 mM KCl, 10 mM Tris–HCl (pH 7.4), 0.1 mM EDTA, 1 mM DTT, 200 μg/ml BSA and 50% glycerol.

### Protein labeling

The cysteine-free variant of *E. coli* RNase H has been successfully used in protein folding and functional studies without affecting its activity (38,39). Therefore, three cysteine residues were replaced with serine or alanine through site-directed mutagenesis (TOYOBO KOD FX Neo) for the site-specific labeling of RNase H with the fluorescent dye (Cy5). Restriction enzyme (Dpn I) treatment was performed, and one cysteine was inserted in the C-terminus. RNase H with the C-terminal cysteine was then expressed and purified (protein purification). RNase H was labeled using a disulfide bond between the thiol group of Cy5 maleimide dye and cysteine on the C-terminus of RNase H. The labeled protein was gently mixed with Ni-NTA agarose resin for 1 h at 4°C. The mixed resin was washed with 15 column volumes (CVs) of buffer containing 20 mM Tris–HCl (pH 7.5) and 500 mM NaCl. Tris(2-carboxyethyl)phosphine (TCEP) (100 μM) was added to the resin, and an excess amount of Cy5-maleimide dye (10 times higher than RNase H) was added to the resin. After the samples on the resin were incubated with slow rotation for 12–14 h at 4°C, free Cy5-maleimide dye was removed by washing with a buffer containing 20 mM Tris–HCl (pH 7.5) and 500 mM NaCl. RNase H labeled with Cy5-maleimide dye was eluted with a buffer containing 20 mM Tris–HCl (pH 7.5), 500 mM NaCl and 100 mM imidazole. After elution, the labeled protein was dialyzed with a buffer containing 20 mM Tris–HCl (pH 7.5) and 150 mM NaCl. The labeled RNase H was stored in buffer containing 50 mM KCl, 10 mM Tris–HCl (pH 7.4), EDTA 0.1 mM, DTT 1 mM, BSA 200 μg/ml and glycerol (50%, v/v).

### Cy3 and Cy5 labeling and annealing

DNA and RNA oligonucleotides were purchased from Integrated DNA Technologies (IDT). Two amine-modified DNA strands were labeled with Cy3 and Cy5, and the nonhydrolyzed strand was constructed by ligating the two oligos using T4 ligase at room temperature for 3 h. The ligated strand was purified from unreacted oligonucleotides via 15% PAGE. The fluorescently labeled nonhydrolyzed DNA was annealed with the 5′ hydrolyzed strand in 10 mM Tris–HCl (pH 8.0) with 50 mM NaCl by heating for 2 min at 85°C and cooling slowly to room temperature. Sequence and modification information is shown in Supplementary Table S1 and structures and abbreviated names of all substrates are provided in Supplementary Table S2 of the supplemental information.

### Single-molecule FRET assays

Single-molecule experiments were performed with a prism-type TIRF (total internal reflection fluorescence) microscope. Briefly, the fluorescence emission light from the donor (Cy3) and acceptor (Cy5) fluorophores was transmitted by a water-immersion objective lens (UPlanApo 60×, Olympus) and then collected through a 550-nm longpass filter to filter out scattered light from a 532 laser. The fluorescence emission spectrum was further divided into donor (green) and acceptor (red) signals with a dichroic mirror with a 630-nm cutoff (Chroma) and recorded by a back-illuminated electron-multiplying charge-coupled device (897 EMCCD, Andor) with the time resolution of 100-ms (for degradation assays) and 500-ms (for binding assays).

### Single-molecule assay of degradation by multiturnover reaction

The substrate was immobilized on the quartz surface covered with PEG through interaction between biotin labels on DNA and Neutravidin (Pierce) on the PEG surface. For single-molecule imaging, we injected ∼10 pM hybrid substrates into the chamber. The reaction buffer contained 50 mM Tris–HCl (pH 8.3), 75 mM KCl, 10 mM MgCl_2_, 100 μg/ml BSA, 1 mg/ml Trolox, 1 mg/ml glucose oxidase, and 0.04% mg/ml catalase. We made the chamber with a coverslip and slide using epoxy and double-sided tape. Holes in the slide were made for the entrance and exit, and a syringe was connected with the holes using tubes. Because the pipette tip containing reaction buffer was fixed to the entrance hole, when the syringe was pulled, the solution in the tip was introduced into the chamber. When RNase H (1, 5, 10, 20, 30, 50, 100 nM) was added to the reaction buffer, the degradation reaction began.

### Single-molecule assay of degradation by single-turnover reaction

The substrate was immobilized in the same way described in the method for the single-molecule assay of degradation by the multiturnover reaction. After immobilization, we injected binding buffer containing 50 mM Tris–HCl (pH 8.3), 75 mM KCl, 1 mM CaCl_2_, 100 μg/ml BSA, 1 mg/ml Trolox, 1 mg/ml glucose oxidase, 0.04% mg/ml catalase, and 5 nM RNase H for binding only. For the removal of free RNase H and a single reaction, we injected washing buffer containing 50 mM Tris–HCl (pH 8.3), 75 mM KCl, 10 mM MgCl_2_, 100 μg/ml BSA, 1 mg/ml Trolox, 1 mg/ml glucose oxidase and 0.04% mg/ml catalase.

### Binding assay with single-molecule FRET

The substrate was immobilized as described in the single-molecule degradation assay by the multiturnover reaction. To observe binding, we used Cy3-labeled substrate and Cy5-labeled RNase H instead of Cy3- and Cy5-labeled substrate and nonlabeled RNase H. We used CaCl_2_ as a binding cofactor to prevent the degradation reaction. Binding buffer containing 50 mM Tris–HCl (pH 8.3), 75 mM KCl, 10 mM CaCl_2_, 100 μg/ml BSA, 1 mg/ml Trolox, 1 mg/ml glucose oxidase, 0.04% mg/ml catalase and 0.2 nM C-terminus Cy5-labeled RNase H was injected into the chamber. We observed binding events through FRET whenever Cy5-labeled RNase H bound to the Cy3-labeled substrate.

### Gel-based degradation assay

Chimeric RNA strands for gel assays were fluorescently labeled via 3′ amino-modification with a Cy3-mono-NHS ester (GE Healthcare Life Sciences). Substrates for the gel assay were prepared as described in Cy3 labeling and annealing. For a degradation reaction, 100 nM substrate was mixed with 5 nM RNase H in 10 μl of buffer containing 10 mM MgCl_2_, 75 mM KCl, 50 mM Tris–HCl (pH 8.3) and 100 μg/ml BSA. The sample was incubated for 20 s (Supplementary Figure S1) and 5 min (Supplementary Figure S14) at room temperature, and the reactions were quenched by the addition of 10 μl of formamide. The result was resolved on a 15% native PAGE gel and imaged by a fluorescence imager (GelDoc, Bio-Rad).

### Quantification and statistical analysis

**Figure 1.**
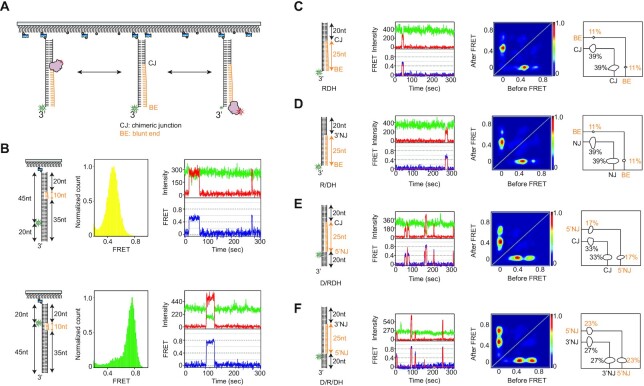
RNase H recognizes diverse junctions flanking RNA:DNA hybrids, regardless of junctional linkage. (**A**) Schematic of the smFRET assay to probe binding dynamics. RNase H and hybrid substrates are labeled with Cy5 and Cy3, respectively. (**B**) Control experiments to assign FRET values using Cy5-labeled RNase H. (top of B) The DNA template strand with the same Cy3 labeling position as shown in C–F was annealed with a 10 nt RNA–20 nt DNA chimeric strand and 35 nt ssDNA, allowing the RNA to act as a single binding site (left cartoon). After Cy5-labeled RNase H was added to the 10 nt RNA region of the hybrid substrate, the FRET value was ∼0.5 (middle, FRET histogram and right, FRET-time trajectory). (bottom of B) When the labeling position was moved close to the RNA region (left cartoon), the FRET value was ∼0.76 (middle, FRET histogram and right, FRET-time trajectory). These control experiments suggested that the binding of RNase H to the Cy3-labeling position of the hybrid substrates used in the main Figures should result in higher FRET, whereas the binding of RNase H to the RNA–DNA chimeric junction should result in lower FRET. (**C**–**F**) Structural variants of RNA:DNA hybrids and binding dynamics of RNase H. (First column) The hybrid substrates (cartoon) are named RDH (C), R/DH (D), D/RDH (E), and D/R/DH (F), where R, D, / and H denote RNA, DNA, nick, and hybrid; (second column) example FRET-time trajectory with fluorescence intensity (top, green for donor and red for acceptor) and FRET efficiency with its idealized guideline (blue and red); (third column) transition density plot (TDP) compiled from binding dynamics; and (fourth column) binding and dissociation frequencies (%, next to circles).

**Figure 2.**
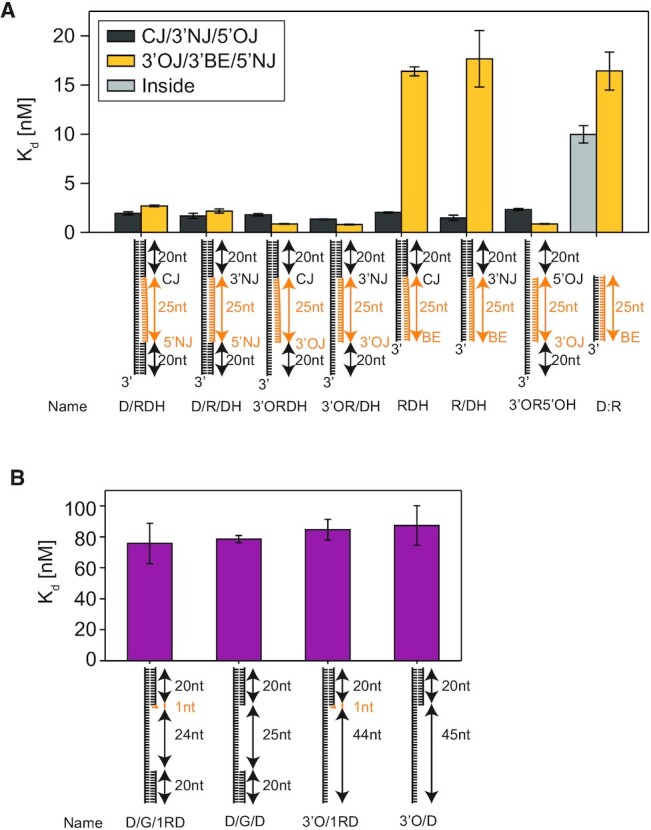
Binding affinities of RNA:DNA hybrid variants. (**A**) Binding affinity (dissociation constant, *K*_d_) is measured using binding and dissociation events occurring between Cy5-RNase H and Cy3-RNA:DNA hybrid variant. *K*_d_ is calculated from [average time of unbound]/[(average time of bound) × (protein concentration)]. (**B**) *K*_d_ is measured by single-molecule binding assays as described in Supplementary Figure S4.

**Figure 3.**
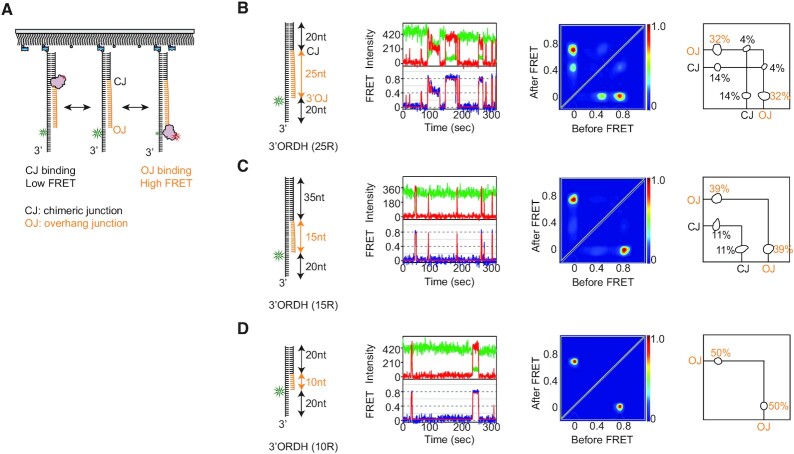
RNase H preferentially recognizes the ss-ds junction bearing a 3′ ssDNA (3′OJ). (**A**) Schematic of the smFRET assay to probe binding dynamics on RNA:DNA hybrids with a 3′ ssDNA overhang. RNase H and its hybrid substrate are labeled with Cy5 and Cy3, respectively. (**B**–**D**) Structural variants of RNA:DNA hybrids and binding dynamics of RNase H. (First column) Three 3′ORDH hybrids whose RNA portion ranged from 25 to 10 bp (cartoon); (second column) example FRET-time trajectory; (third column) transition density plot (TDP) compiled from binding dynamics and (fourth column) binding and dissociation frequencies (%, next to circles).

**Figure 4.**
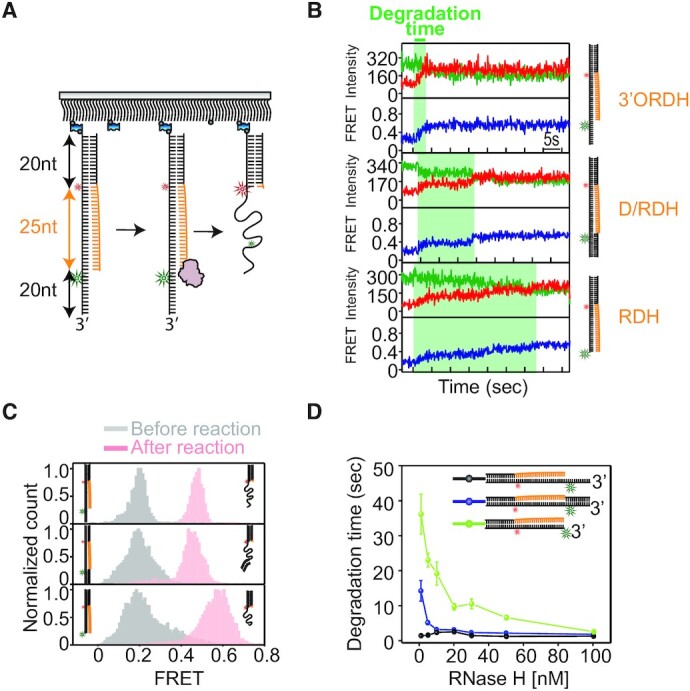
smFRET assay of RNA degradation by RNase H. (**A**) Schematic of the smFRET assay obtained from the multiturnover reaction (MR) to quantify RNA degradation kinetics. The experiments were performed by adding a solution containing Mg^2+^ and RNase H to hybrid substrates. (**B**) Representative FRET-time trajectories obtained from 3′ORDH (top), D/RDH (middle) and RDH (bottom). The green region shows how degradation times are measured. (**C**) FRET histograms before (gray) and after the reaction (pink) obtained from 3′ORDH (top), D/RDH (middle) and RDH (bottom). (**D**) Average degradation time versus RNase H concentration (1–100 nM). Error bars denote s.e.m. unless otherwise indicated.

**Figure 5.**
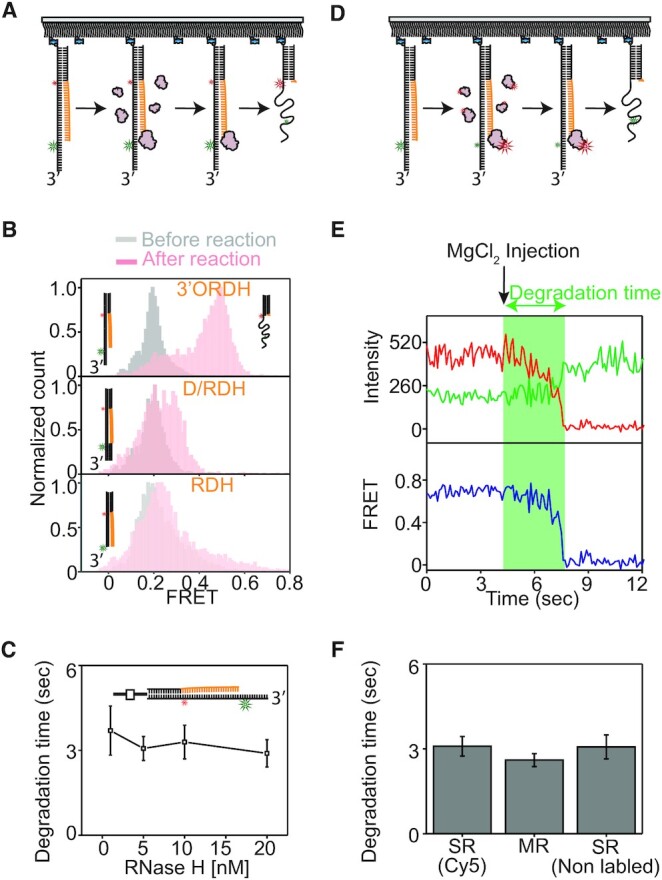
In the presence of a 3′ ssDNA overhang on RNA:DNA hybrids, RNase H becomes a processive exoribonuclease degrading RNA oligonucleotides in the 5′ to 3′ direction via tight anchoring at the ss-ds overhang junction. (**A**, **D**) Schematic of the smFRET assay for single-turnover degradation reactions (SR) performed by flowing in 10 mM Mg^2+^ buffer for various substrates (3′ORDH, D/RDH, and RDH). The flow removes free proteins in solution liberated from the substrates, which prevents RNA degradation caused by the rebinding of free proteins from the solution. This assay allows us to examine whether the enzyme performs processive degradation per binding event. (**B**) FRET histograms before (gray) and after the reaction (pink) obtained from SR at varying substrates (3′ORDH, D/RDH, and RDH). (**C**) Degradation time versus RNase H concentration, showing enzyme concentration-independent degradation obtained from SR using unlabeled RNase H and Cy3-Cy5-3′ORDH. (**E**) Representative FRET-time trajectory obtained from the SR of Cy3-3′ORDH and Cy5-RNase H. The green region shows how the degradation time is measured. (**F**) Comparison of the degradation times of 3′ORDH between SRs by a single binding event and MRs by multiple binding and dissociation events at 5 nM RNase H (e.g. first bar from Figure 5E; second bar from black of Figure 4D and third bar from Figure 5C).

**Figure 6.**
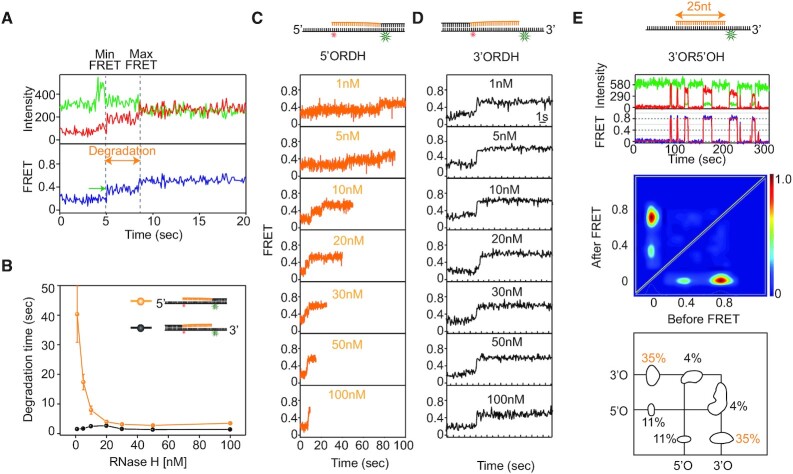
Comparison of the enzymatic mechanism of action of RNase H in the presence of 3′ and 5′ ssDNA overhangs on RNA:DNA hybrids. (**A**) Representative FRET-time trajectory with fluorescence intensity (top, green for donor and red for acceptor) and FRET efficiency (bottom, blue). The vertical dotted lines indicate the degradation period during which FRET increases from the minimum to the maximum values, and the green arrow indicates the pause due to dissociation and rebinding of RNase H. The MR experiments were performed by adding a solution containing Mg^2+^ and RNase H to the hybrid substrate. (**B**) Comparison of degradation times between 5′ORDH (orange) and 3′ORDH (black) as a function of RNase H concentration. (**C**) Representative FRET-time trajectories at varying protein concentrations for 5′ORDH. (**D**) Representative FRET-time trajectories at varying protein concentrations for 3′ORDH. (**E**) Direct comparison of binding frequencies between two ss-ds junctions with overhangs of 5′ ssDNA (low FRET, *E* = 0.38) and 3′ ssDNA (high FRET, *E* = 0.78). Representative FRET-time trace (top), TDP (middle) and binding frequency (bottom) are displayed. TDP shows a higher binding frequency at the junction with the 3′ overhang (3′OJ, 35%) than at the junction with the 5′ overhang (5′OJ, 11%).

**Figure 7.**
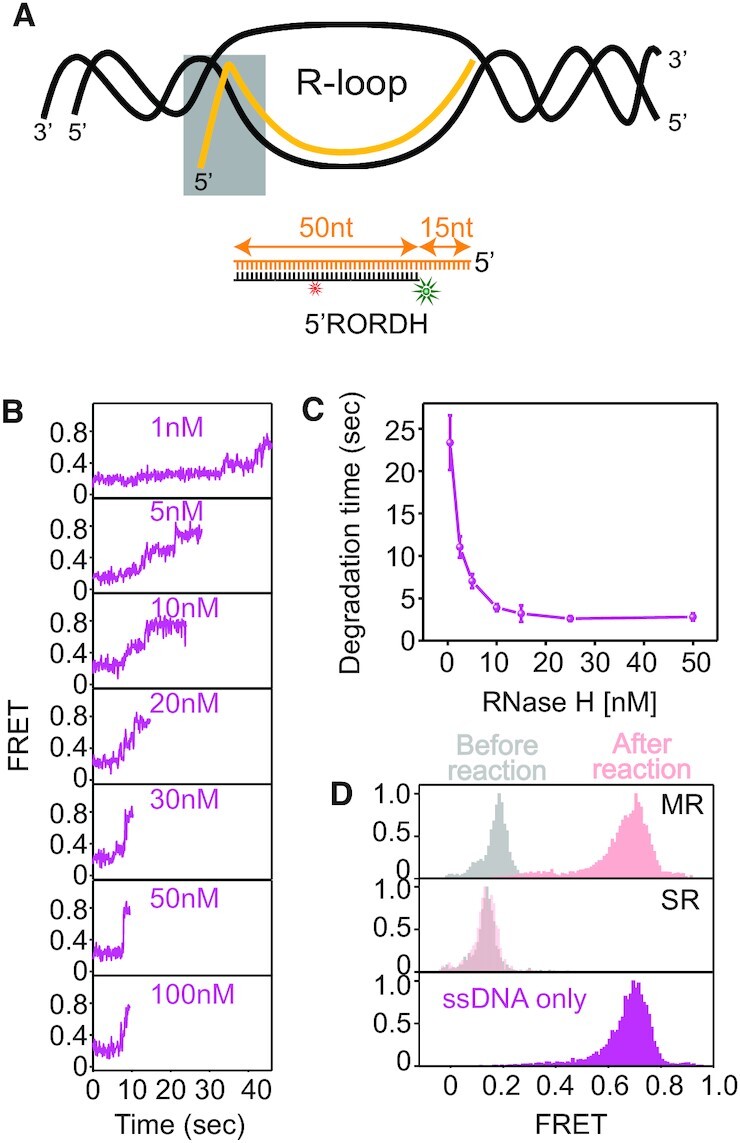
Degradation kinetics of an RNA:DNA hybrid with a 5′ ssRNA overhang (5′RORDH) present in R-loops. (**A**) Schematic of R-loop, containing a 5′ ssRNA overhang and RNA:DNA heteroduplex (gray box). (**B**) Representative FRET-time trajectories at varying protein concentrations for 5′RORDH under 10 mM Mg^2+^. (**C**) Degradation time versus RNase H concentration, showing protein concentration-dependent degradation obtained from MR using unlabeled RNase H and Cy3-Cy5-3′RORDH. (**D**) Comparison of FRET histograms obtained from MR (top), SR (middle), and ssDNA only (bottom): before (gray), after the reaction (pink) on 5′RORDH, and ssDNA only mimicking the degradation product (magenta) under 5 nM RNase H and 10 mM Mg^2+^.

The average time, count, frequency, running time, and fraction were generated from at least three repeated experiments (n). The errors of degradation time, count, frequency, running time, and fraction indicate the standard error of the mean (s.e.m.): s.e.m. = σ/√*n* (Figures 2, 4D, 5C, 5F, 6B, 7C, Supplementary Figure S2B, S3B, S3C, S4, S5B, S6C, S8A, S9A, S9B, S10B, S12 and S13A). The percentage of each area in TDP (next to circles) was generated from (number of events in selected area)*100/(number of events in all areas), which was mentioned as the binding probability (Figures 1C–F, 3B–D, 6E and Supplementary Figure S12A). The statistics of all figures is given in Supplementary Table S3 of the supplemental information.

## RESULTS

### Single-molecule fluorescence binding assay for RNase H

To perform binding assays at the single-molecule level (Figure 1A), *E. coli* RNase H was site-specifically labeled with a Cy5 acceptor (termed Cy5-RNase H) at the C-terminus via cysteine engineering (40), and the labeling did not significantly alter the enzymatic activity (Supplementary Figure S1). Complementarily, the four hybrids were labeled with a donor (Cy3) on the nucleotide of the DNA strand opposite to the 5′ end of the ssRNA (each termed Cy3-‘substrate name’), and the hybrid substrates were tethered to a polymer-coated surface via a biotinylated duplex end (Figure 1A). For the RNA:DNA hybrid substrates, DNA and RNA are shown in black and orange.

To assign FRET values for binding sites, the DNA template strand with the same Cy3 labeling position was annealed with a 10 nt RNA–20 nt DNA chimeric strand and 35 nt ssDNA, allowing the RNA to act as a single binding site (top, left of Figure 1B). After Cy5-labeled RNase H was bound to the 10 nt RNA region of the hybrid substrate, the FRET value was ∼0.5 (top, middle and right). When the labeling position was moved close to the RNA region (bottom, left of Figure 1B), the FRET value was ∼0.76 (bottom, middle and right). These control experiments suggested that the binding of RNase H to the Cy3-labeling position of the hybrid substrate should result in higher FRET, whereas the binding of RNase H to the RNA–DNA chimeric junction should result in lower FRET.

To investigate whether discontinuities in RNA:DNA hybrids served as recognition sites, we constructed four RNA:DNA hybrids (first column in the left of Figure 1C–F), one of which (Figure 1E) occurs physiologically when DNA polymerase fills gaps between Okazaki fragments (41). The hybrids contained structural discontinuities, e.g. an RNA–DNA chimeric junction (CJ) without a nick, a blunt end (BE), a nick junction on the 3′ side of ssRNA (3′NJ) in the chimeric strand, and a nick junction on the 5′ side of ssRNA (5′NJ). The RNA:DNA hybrids were named RDH, R/DH, D/RDH, and D/R/DH (first column in the left of Figure 1C–F), where R, D, / and H denote RNA, DNA, nick and hybrid, respectively. The constructs contained a 20 base pair (bp) DNA:DNA duplex on one side (RDH and R/DH) or on both sides (D/RDH and D/R/DH) flanking a 25 bp RNA:DNA hybrid. Since the DNA:DNA duplex and RNA:DNA hybrid had the B-form and A-form conformations, respectively, a structural discontinuity occurred at the junctions (i.e. CJ, 3′NJ and 5′NJ).

### Binding modes of various RNA:DNA hybrids without ssDNA overhangs determined by smFRET

Upon the addition of Cy5-RNase H to Cy3 hybrids, we found that RNase H did not stably bind to the hybrids in the absence of metal ions at RNase H concentrations at 0.2 nM (Supplementary Figure S2). Metal ions are known to serve as binding cofactors in DNA-protein interactions (24,42,43). To monitor substrate recognition by RNase H without allowing the reaction to proceed, Ca^2+^ was used as a catalytically inactive ion (24,43), and 10 mM Ca^2+^ was found to be a suitable condition based on a test of the dependence on divalent ion concentration (Supplementary Figure S3). According to structural and computational studies (20,22,43,44), the binding modes of RNase H with Ca^2+^ are not notably perturbed compared to those with Mg^2+^, except for a slight alteration in the metal ion coordination. All binding and dissociation events were thus monitored in the presence of 10 mM Ca^2+^ and pictorialized into a transition density plot (TDP), which recapitulated the two-dimensional histogram for the pair of FRET values before and after each binding event (Figure 1C–F). For the RDH and R/DH hybrids, low and high FRET were detected at *E* = 0.48 and 0.77, and the dominant binding sites were the CJ and 3′NJ at *E* = 0.48, regardless of the presence of a nick (C and D in Figure 1). The preferential binding to the chimeric junction for RDH was consistent with the findings of a previous study (23,45–47). For D/RDH, the binding of CJ at low FRET was more favorable than that of 5′NJ at high FRET (Figure 1E), but for D/R/DH, the binding at low and high FRET was comparable when both nicks were present (Figure 1F). Overall, we found that RNase H recognized various structural discontinuities at the CJ, 3′NJ and 5′NJ but not very well at the BE and the inner region of RNA:DNA hybrids (Figure 1C-F).

### Affinities of RNA:DNA hybrid variants and degradation products

For Cy5-RNase H and eight Cy3-RNA:DNA hybrid variants (cartoons in Figure 2 and Supplementary Table S2), binding and dissociation events were recorded, and their binding affinities (K_d_) were attained from [average time of unbound]/ [(average time of bound) × (protein concentration)] (48,49). Their binding affinity was ranked as follows (Figure 2): 3′ ssDNA-overhang-containing hybrids, on the order of ∼0.8 nM (e.g. 3′ORDH, 3′OR/DH and 3′O5′ORDH); structural duplex discontinuities, ranging from ∼1.3 to 2.7 nM (e.g., CJ, 3′NJ and 5′NJ); the inner regions of hybrids, on the order of ∼10 nM (e.g. RNA:DNA); and the blunted ends of hybrids, on the order of ∼16 nM (e.g. RDH, R/DH and RNA:DNA). Furthermore, we measured the affinities of the degradation products (Figure 2B and Supplementary Figure S4). The affinities of the four degradation products were very similar, ranging from ∼75 nM to ∼87 nM, even for the products containing a single ribonucleotide (D/G/1RD and 3′O/1RD). The comparable result in Figure 2B is in good agreement with the fact that recognition by RNase H requires a minimum of four consecutive ribonucleotides (14,22).

### The 3′ ssDNA-overhang junction is an unprecedented recognition site for RNase H

Overhangs of 3′ ssDNA in RNA:DNA hybrids are created and grown during the degradation of the R-loop (3), and also appear early when DNA synthesis is not completed to the 5′ end of the next RNA primer during the synthesis of lagging strands (50). To examine how the ssDNA overhang influences the degradation kinetics of RNase H, a 3′ ssDNA overhang 20 nucleotides (nt) long was attached to three 3′ORDH hybrids whose RNA portion ranged from 25 to 10 bp (left of Figure 3B–D). Upon the addition of Cy5-RNase H, all binding events in the presence of Ca^2+^ were plotted to TDP for the hybrids carrying the 3′ ssDNA overhang. For the 3′ overhang-bearing RDH (i.e. 3′ORDH), the primary binding site appeared at the ss-ds junctions (termed 3′OJ) consisting of a 3′ ssDNA overhang and RNA:DNA heteroduplex, represented by the high FRET, *E* = 0.77, and the secondary binding site appeared at the CJ, represented by the low FRET, *E* = 0.48 (Figure 3B). The difference in binding events at the 3′OJ and at the CJ (TDP in Figure 3B) was consistent with the *K*_d_ values of ∼0.8 and ∼1.8 nM, respectively (Figure 2A). Interestingly, given two robust binding sites, ∼8% of RNase H was able to diffuse (FRET-time trajectories and rates in Supplementary Figure S5), scanning approximately 25 bp along the substrate without hydrolyzing RNA (4% and 4%, right panel in Figure 3B).

As the length of the RNA:DNA portion decreased while the length of the 3′ ssDNA overhang was maintained, the high FRET at the 3′OJ binding site became predominant, and the low FRET at the CJ disappeared (Figure 3B–D). This observation confirmed that the high FRET indeed resulted from binding to the 3′OJ, which was the Cy3-labeling position. Although the binding of RNase H to CJ was previously known (16,51), the binding to the 3′OJ was not previously known. In particular, most structural and biochemical studies have used RNA:DNA hybrids (termed D:R) without any flanking ssDNA and dsDNA. We then examined the binding of RNase H to a 25 bp D:R and found no notable binding site along the duplex axis (Supplementary Figure S6A and B). Furthermore, the 3′OJ binding affinity of the 3′ORDH was ∼11–20 times higher than the affinity of D:R (Figure 2A and Supplementary Figure S6C). We therefore conclude that the previously unidentified 3′OJ serves as the predominant binding site for RNase H if the hybrid substrate contains at least 10 bp RNA (Figure 3B–D).

### Assay of multiturnover RNA degradation by RNase H

To unravel the role of the 3′OJ during the enzymatic reaction, the degradation activity of RNase H in the presence of Mg^2+^ was monitored in real time. The 3′ORDH substrate, containing a 20 nt 3′ overhang, a 25 bp RNA:DNA hybrid, a CJ, and a 20 bp dsDNA, was labeled with a FRET pair in which the donor (Cy3) and acceptor (Cy5) were conjugated at the DNA sites opposite the 5′ end of ssRNA and the CJ of the RNA–DNA strand, respectively (Figure 4A). We used smFRET to examine how the 3′ overhang participates in the RNA degradation of three hybrid substrates, 3′ORDH, D/RDH and RDH (right cartoons in Figure 4B). When a reaction solution containing RNase H and 10 mM MgCl_2_ was added to the hybrid substrates, RNA degradation took place, converting the duplex hybrid to ssDNA. RNA degradation decreased the time-averaged distance between the two fluorophores, leading to an increase in FRET for all three substrates. In the FRET-time trajectories, the degradation reaction gave rise to progressive increases in FRET over time (Figure 4B). After RNA degradation, all three substrates showed high FRET ranging from 0.46 to 0.58 (Figure 4C), consistent with the control experiments with degradation-mimic substrates (Supplementary Figure S7). For this type of experiment, we coined the term ‘multiturnover reaction’ (MR) because many binding and dissociation events could occur during degradation by free RNase H in solution, liberated from the substrates.

To characterize the kinetics of degradation by RNase H, the degradation time was measured as a function of protein concentration, ranging from 1 nM to 100 nM. Surprisingly, the duration of degradation showed different degrees of dependence on protein concentration. The 3′ORDH exhibited protein concentration independence (black in Figure 4D), whereas the other two hybrids without the 3′ ssDNA overhang (D/RDH and RDH) showed strong protein concentration dependence (blue and green in Figure 4D). Close examination of individual FRET-time trajectories showed a gradual decrease in degradation time at higher protein concentrations (Supplementary Figure S8). This protein concentration dependence has been classified as indicating a distributive enzymatic activity (31), which means that the degradation time becomes longer if the enzyme is prone to dissociation simply because RNA degradation ceases until RNase H rebinds to the RNA:DNA substrate at low concentrations. Taken together, these results suggested that the 3′ ssDNA overhang facilitates processive degradation by stabilizing the binding of RNase H at the 3′OJ in 3′ORDH with sub-nanomolar affinity (Figures 2A and 3B).

### 3′OJ facilitates the processive degradation of RNA oligonucleotides by RNase H

To reconfirm whether the 3′OJ promoted RNase H to processively degrade RNA in the hybrid, we then performed a so-called ‘single-turnover reaction’ (SR), which allowed us to measure the degree of degradation per attempt. The RNA hybrid was incubated with RNase H (5 nM) in the presence of Ca^2+^ for 5 min, which permitted RNase H to bind, and then the reaction was started by flowing in the reaction buffer with 10 mM Mg^2+^. The flow flushed free proteins out in solution, preventing rebinding of RNase H (Figure 5A) so that only prebound RNase H contributed to the reaction. This assay allowed us to inspect the mode of enzymatic degradation, such as processive degradation by a single binding event, distributive degradation by multiple binding events, or a combination of both. Based on FRET histograms (Figure 5B), the SR of 3′ORDH resulted in complete degradation (top panel), whereas the SR of D/RDH and RDH without the 3′ ssDNA overhang showed incomplete degradation (middle and bottom panels). The analysis of 3′ORDH degradation by SR revealed no dependence on protein concentration (Figure 5C), indicating that the enzyme performed processive degradation on 3′ORDH but not on D/RDH and RDH, which lacked the 3′ overhang. Furthermore, the protein concentration independence was maintained even at a rate 4-fold slower than the standard degradation rate of RNase H, confirming the processive degradation (Supplementary Figure S9).

To directly validate the processive degradation, we visualized the enzymatic action during the SR of RNA degradation using Cy5-RNase H and Cy3-3′ORDH with a 3′ overhang (Figure 5D). Upon the addition of 10 mM Mg^2+^, the prebound Cy5-RNase H processively degraded 3′ ORDH while moving away from its Cy3 position, progressively decreasing FRET without any signal loss (Figure 5E). If RNase H had been distributive and dissociated, we would have observed such a dissociation event as a sudden loss of the fluorescence signal before the FRET decrease was complete. We analyzed the degradation times of three different reactions (i.e. SR to Cy5-labeled RNase H; MR to nonlabeled protein; and SR to nonlabeled in Figure 5F). Their degradation times were comparable, confirming that RNase H performs processive degradation in the presence of the 3′OJ carrying the 3′ ssDNA overhang. In contrast, the scrutiny of degradation products after SR by Mg^2+^, using the binding event patterns in the presence of Ca^2+^, showed that Cy3-D/RDH was not completely degraded due to distributive degradation, but Cy3-3′ORDH with the 3′-overhang was completely degraded by the processive degradation of nonlabeled RNase H (Supplementary Figure S10)

### RNase H is a ribonuclease with asymmetric directionality depending on the type of ssDNA overhang

To investigate the effect of the opposite 5′ overhang, the degradation times of 5′ORDH and 3′ORDH were compared (Figure 6A–D). Analysis of FRET-time trajectories by MR showed protein concentration-dependent degradation of 5′ORDH (orange in Figure 6B) but protein concentration-independent degradation of 3′ORDH (black in Figure 6B). Close inspection of individual FRET-time trajectories of 5′ORDH showed pauses during degradation (indicated by green arrows in Figure 6A), indicating dissociation and rebinding of RNase H. Unlike that of 3′ORDH, the SR on 5′ORDH showed incomplete degradation (Supplementary Figure S11). Again, concentration dependence is evidence of distributive degradation, but concentration independence is evidence of processive degradation, as previously proposed (31). Furthermore, we directly compared the binding difference between the 3′OJ and 5′OJ using Cy5-RNase H and Cy3-3′OR5′OH bearing both 3′ and 5′ ssDNA overhangs, each 20 nt long (top in Figure 6E). The binding probability of the 3′OJ site was clearly ∼3 times higher than that of the 5′OJ site (bottom of Figure 6E), and the affinities (*K*_d_) of the 3′OJ and 5′OJ were ∼0.87 and 2.34 nM, respectively, consistently showing a difference of ∼3-fold (3′OR5′OH in Figure 2A).

To clarify the nature of enzymatic directionality and activity on the 5′ORDH, binding and degradation assays were performed (Supplementary Figure S12). The *K*_d_ values of 5′OJ and CJ were similar for both junctions of 5′ORDH, with ∼2.23 and ∼1.76 nM, respectively (Supplementary Figure S12A). The average binding (on) and dissociation (off) times at 10 nM Cy5-labeled RNase H were ∼0.23 and ∼6.56 s, respectively (right in Supplementary Figure S12B). The average pause time was ∼7.65 s at 10 nM nonlabeled RNase H (green arrow in Figure 6A and top-left in Supplementary Figure S12B). The similarity between dissociation and pause times (i.e. ∼6.56 and ∼7.65 s, respectively) indicates that RNase H dissociates from the 5′ORDH when the degradation reaction is paused, suggesting distributive degradation. In summary, RNase H is a ribonuclease with asymmetric directionality that performs different degradation kinetics depending on whether RNA:DNA hybrids possess a 3′ or 5′ ssDNA overhang.

To reconfirm that RNase H binds to the 3′ OJ, a 2-bp mismatch was introduced to the 5′ end of RNA at the 3′OJ of 3′ORDH, and the degradation time was measured (inset in Supplementary Figure S13A). The plot of degradation time versus enzyme concentration showed that the degradation of 3′ORDH with the 2-bp mismatch was slower than the degradation of intact 3′ORDH. Based on the clear effect of the 2-bp mismatch at the 3′OJ on the ssDNA overhang in RNA:DNA hybrid, we concluded that RNase H preferentially bound to the 3′OJ and degraded RNA but not the inner region of RNA in the presence of 3′ ssDNA overhangs. Otherwise, the delay in degradation due to the melting effect with the 2-bp mismatch at the 3′OJ would not have occurred (Supplementary Figure S13).

Like ssDNA overhangs, 5′ ssRNA overhangs can also be generated in R-loops (gray box in Figure 7A). We used a RNA:DNA hybrid with a 5′ ssRNA overhang (5′RORDH, cartoon of Figure 7A). Degradation time was measured as a function of RNase H concentration, ranging from 1 to 100 nM (Figure 7B and C). FRET-time trajectories showed a gradual decrease in the time-interval required for degradation from low FRET (*E* = 0.2) to high FRET (*E* = 0.7) with increasing enzyme concentration (Figure 7B). This protein concentration dependence indicates a distributive enzymatic activity (Figure 7C). To reconfirm the distributive mode of enzymatic action, the SR was performed. Indeed, the reaction did not result in complete degradation (middle in Figure 7D). We concluded that RNase H performs distributive degradation on the hybrid with a 5′ ssRNA overhang.

### A model of RNase H activity

**Figure 8.**
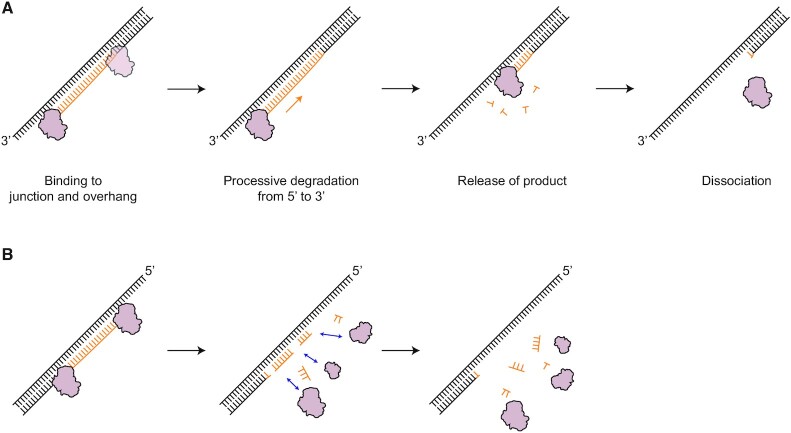
Model of the degradation mechanism of action of RNase H, in which its directionality and kinetics are determined by the type of overhang of RNA:DNA hybrids. (**A**) In the presence of a 3′ ssDNA overhang on RNA:DNA hybrids, RNase H becomes a processive exoribonuclease that continuously degrades RNA in the 5′ to 3′ direction via tight anchoring at the 3′OJ. (**B**) In the presence of a 5′ ssDNA overhang or in the absence of any 3′ and 5′ overhangs on the RNA:DNA hybrids, RNase H functions as a sequence-nonspecific endoribonuclease, randomly degrading RNA moieties of RNA:DNA hybrids in a distributive manner. All of this makes RNase H a multifunctional enzyme with asymmetric directionality. The processive exonuclease activity in the 5′ to 3′ direction is the primary physiological degradation mode if 3′ ssDNA overhangs are present during RNA elimination by RNase H.

Based on all the smFRET and biochemical data, we propose a model in which RNase H acts as an endo- and exoribonuclease with random and unidirectional enzymatic action, respectively (Figure 8). The directionality and kinetics are thus determined by the type of ssDNA overhangs of RNA:DNA hybrids. In the presence of a 3′ overhang, RNase H processively degrades the RNA moieties of RNA:DNA hybrids without falling off the substrates (Figure 5E and F), whereas in the absence of a 3′ overhang (e.g. RDH, D/RDH and 5′ORDH), it degrades RNA with a nondirectional and distributive mode of action in its previously known role as a non-sequence-specific endonuclease, undergoing multiple binding and dissociation events (Figure 6B–C and Supplementary Figure S8C). In this model, we draw degradation products as mono-ribonucleotides as in a typical exonuclease (31,52) and arbitrary sizes as in sequence-nonspecific endoribonucleases (46,53).

The presence of the 3′ ssDNA overhang provides a stable binding site for RNase H at the 3′ partial RNA:DNA heteroduplexes, allowing robust processivity. We speculate that flexible 3′ ssDNA forms a protein-nucleic acid complex as if it wraps around the positively charged surface of RNase H. The fact that RNase H can interact stably with the 3′OJ but not with the 5′OJ (Figure 6E) suggests that RNase H probes and utilizes ‘junctional polarity’ via ssDNA overhangs. The structural polarity of the 3′ and 5′ overhangs may direct the appropriate RNA-processing functions at Okazaki fragments and R-loops, indicating the need for either processive or distributive degradation.

## DISCUSSION

We found that various discontinuities (e.g. CJ, OJ and NJ sites) in RNA:DNA hybrids function as structural recognition sites for RNase H (Figures 1 and 3). The RNA:DNA hybrid in complex with RNase H has been shown to adopt a mixed A and B duplex form (14,54), where the RNA strand is in the A-form with C3′-endo sugar puckers, whereas the DNA strand is in the B-form with C2′-endo sugar puckers. This structural feature allows RNase H to distinguish the RNA:DNA hybrids from dsRNA and dsDNA (54). Although the binding at the CJ by RNase H2 has been structurally resolved (16), the ability of RNase H to bind to the NJ and OJ has been newly discovered. In particular, dsDNA and ssDNA junctions flanking RNA:DNA hybrids serve as recognition sites with ∼40–100-fold higher affinity than ss-dsDNA junctions (Figure 2). The binding preference for structural discontinuities at flanking junctions may help RNase H find RNA:DNA hybrids in long genomic DNA.

The functional role of the 3′OJ has not been previously identified, presumably due to a lack of structural and dynamic data between RNase H and 3′ overhang-bearing RNA:DNA hybrids. Thanks to the direct visualization of RNase H activity, we have discovered the previously unknown role of the 3′OJ in enabling RNase H to perform processive degradation. RNase H stably bound to the 3′OJ via a tight grip or recognition with ∼11 times more tightly (i.e. lower *K*_d_) than to the inner region of RNA:DNA hybrids (3′ORDH and D:R in Figure 2A and Supplementary Figure S6). The resulting processivity did not occur on blunt-ended hybrids without any overhangs or on hybrids with a 5′ overhang.

The incorporation of ribonucleotides into the genome is known to be a genotoxic element, leading to genomic instability in both eukaryotes and bacteria (1,2,55,56). The R-loops vary from a few hundred bp to a few kilobases in length (57), which suggests that RNase H should be able to perform processive and robust activity to remove RNA from stable RNA:DNA heteroduplexes. In eukaryotes, RNase H1 has evolved to adopt the framework of HBD for processive function (26)^,^ whereas RNase H2 has become a trimeric enzyme and interacts with PCNA for processive function (58). However, the mechanism of processivity of RNase HI in bacteria was formerly unknown. We found that RNase HI possesses the intrinsic ability to perform processive degradation in the presence of 3′ ssDNA overhangs in RNA:DNA hybrids. Then, what is the biological implication of the processive activity of RNase H? Since proteins, e.g. a lac repressor, take 1–5 min to find the target site (59), it can be difficult for RNase H to find small stretches of RNA within a limited time in *E. coli*, where cells divide every 20 min and number of Okazaki fragments are ∼2300–4600. Therefore, processive degradation of RNase H on 3′ ssDNA might be beneficial for the complete DNA replication in a timely manner, compared to distributive degradation.

RNase H has long been known to be an endonuclease that nonspecifically hydrolyzes RNA moieties in RNA:DNA hybrids. How can our finding be reconciled with the previous crystal structures showing the binding complex of RNase H with RNA:DNA hybrids in the inner region of the hybrids? Our smFRET assays of D:R and D/RDH clearly showed that RNase H cleaves RNA randomly in the inner region of hybrids (Supplementary Figures S6B and S10C) when ssDNA overhangs are absent or hybridized by the complementary strand. In addition, the TDP of D:R (Supplementary Figure S6) showed the binding mode of RNase H toward its inner region in the absence of ssDNA overhangs, consistent with the structural results. However, the inner region of RNA:DNA hybrids is not the dominant binding site for RNase H in the presence of ssDNA overhangs or various heteroduplex junctions (*K*_d_ in Figure 2A). Therefore, our data and previous reports in combination can expand the known mechanism of RNase H, a non-sequence-specific endoribonuclease, to a versatile asymmetric endo- and exo-like ribonuclease that recognizes the overhang polarity of RNA:DNA hybrids (Figure 6) and performs different modes of degradation (Figure 8). However, the structural binding mode for processive degradation needs to be further investigated at the atomistic level by X-ray crystallography, NMR, and cryo-electron microscopy.

In short, we developed smFRET assays to probe the functional roles of various structural features along linear RNA:DNA hybrids (e.g. CJ, NJ, 3′OJ and 5′OJ). Our study offers novel mechanistic insights into the recognition of RNase H in RNA:DNA intermediate structures containing dsDNA flanking junctions. We also found that the 3′ ssDNA overhang, if present, facilitates the overall reaction, making RNase H a processive exoribonuclease. Furthermore, RNase H successively degrades RNA while translocating along the RNA:DNA hybrid without falling off. This asymmetric bifunctional enzymatic activity makes RNase H unique in efficiently removing RNA moieties during dynamic replication, transcription, and repair processes. During nucleic acid metabolism, 3′ overhang and 5′ overhang strands are frequently produced by various nucleases, suggesting that this type of interaction may occur with other DNA- and RNA-interacting proteins, allowing these nucleases to perform a variety of functions.

## DATA AVAILABILITY

The data that support the findings of this study are available from the corresponding author upon reasonable request.

## Supplementary Material

gkab1064_Supplemental_File
