# Supplementary material for: RNase H is an exo- and endoribonuclease with asymmetric directionality, depending on the binding mode to the structural variants of RNA:DNA hybrids
Source: Nucleic Acids Res. 2021 Nov 12;50(4):1801–14. doi: 10.1093/nar/gkab1064 (PMC8886854; doi:10.1093/nar/gkab1064)

Supplemental Information

RNase H is an exo- and endoribonuclease with asymmetric directionality, depending on the binding mode to the structural variants of RNA:DNA hybrids

Hyunjee Lee, HyeokJin Cho, Jooyoung Kim, Sua Lee, Jungmin Yoo, Daeho Park, and Gwangrog Lee

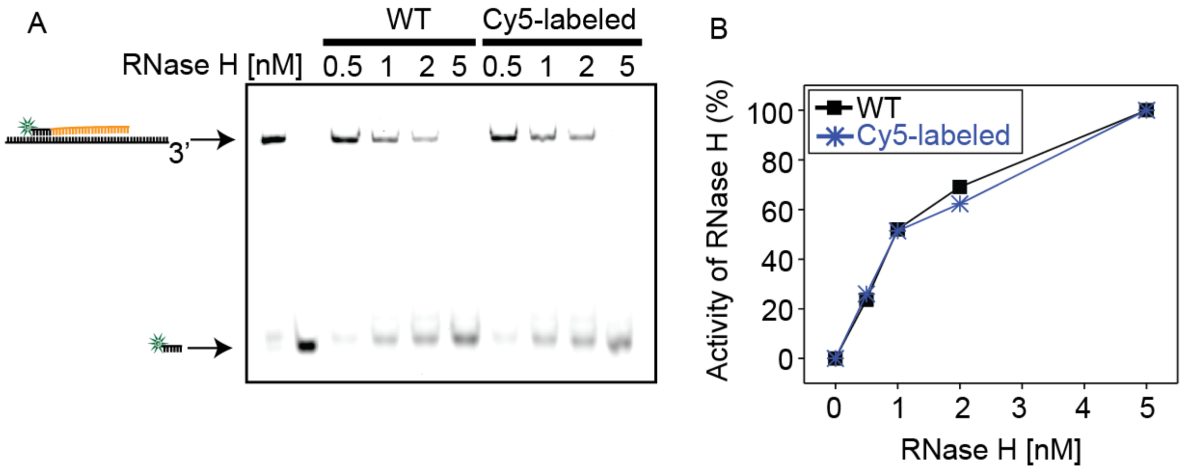

Figure S1. C-terminal labeling of RNase H using cysteine engineering and control degradation experiments for the labeling effect.

(A) Gel assay for degradation by RNase H to confirm that the labeling of RNase H does not significantly affect the activity of RNase H. The 3' end of chimeric RNA-DNA was labeled with Cy3 and annealed with nonlabeled DNA template (left cartoon). The substrate was incubated with each nonlabeled and Cy5-labeled RNase H for 20 s at room temperature. To stop the reaction, formamide was added, and the gel was imaged using a fluorescence imager (ChemiDoc^{Tm} MP System, Bio-Rad). (B) Degradation activity as a function of RNase H concentration obtained from the quantification of the product bands using ImageLab^{TM} software.

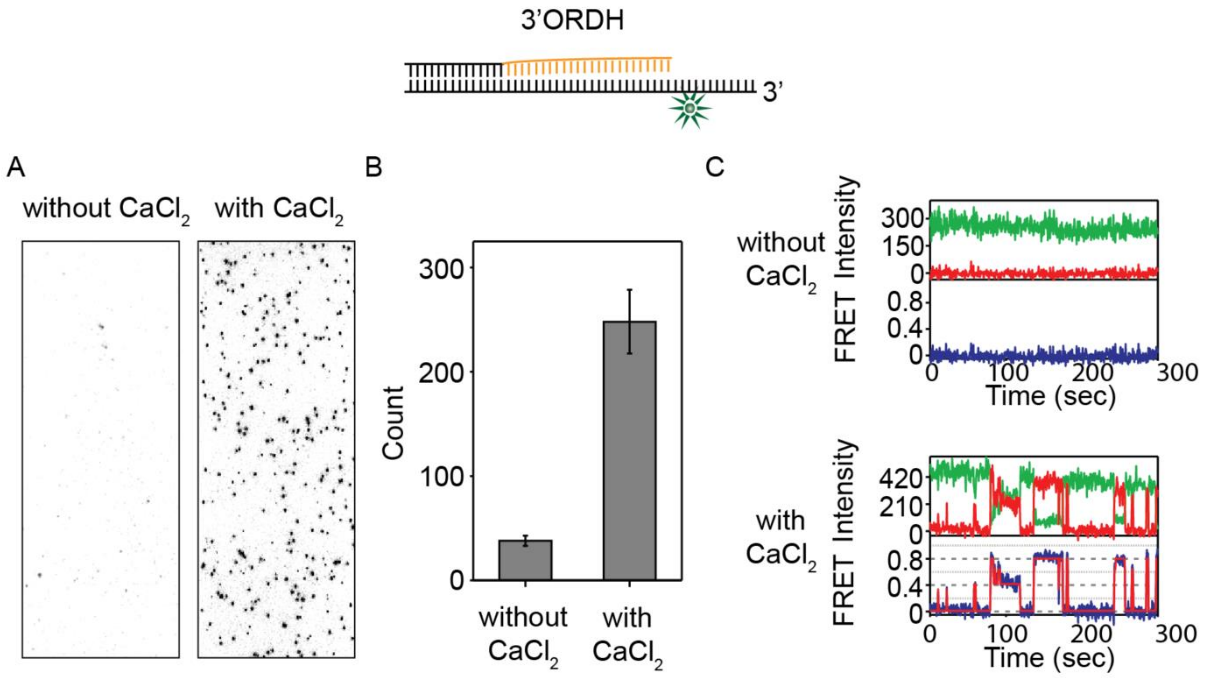

Figure S2. In the absence of metal ions, RNase H cannot bind to RNA:DNA hybrids, indicating that metal ions serve as binding cofactors for nucleic acid-protein interactions.

(A) The binding assay in the absence and presence of Ca^{2+}. Cy5-labeled RNase H was added to the Cy3-3’ORDH substrate immobilized on the imaging surface, and the binding complexes of 3’ORDH and RNase H were counted by Cy5 excitation without $CaCl_2$ (left) and with $CaCl_2$ (right). (B) Binding counts of protein-substrate complexes without $CaCl_2$ (38±4.85) and with $CaCl_2$ (248±30.55, with s.e.m.). (C) Representative FRET-time trajectory obtained from binding events between 3’ORDH and RNase H without $CaCl_2$ (top) and with $CaCl_2$ (bottom).

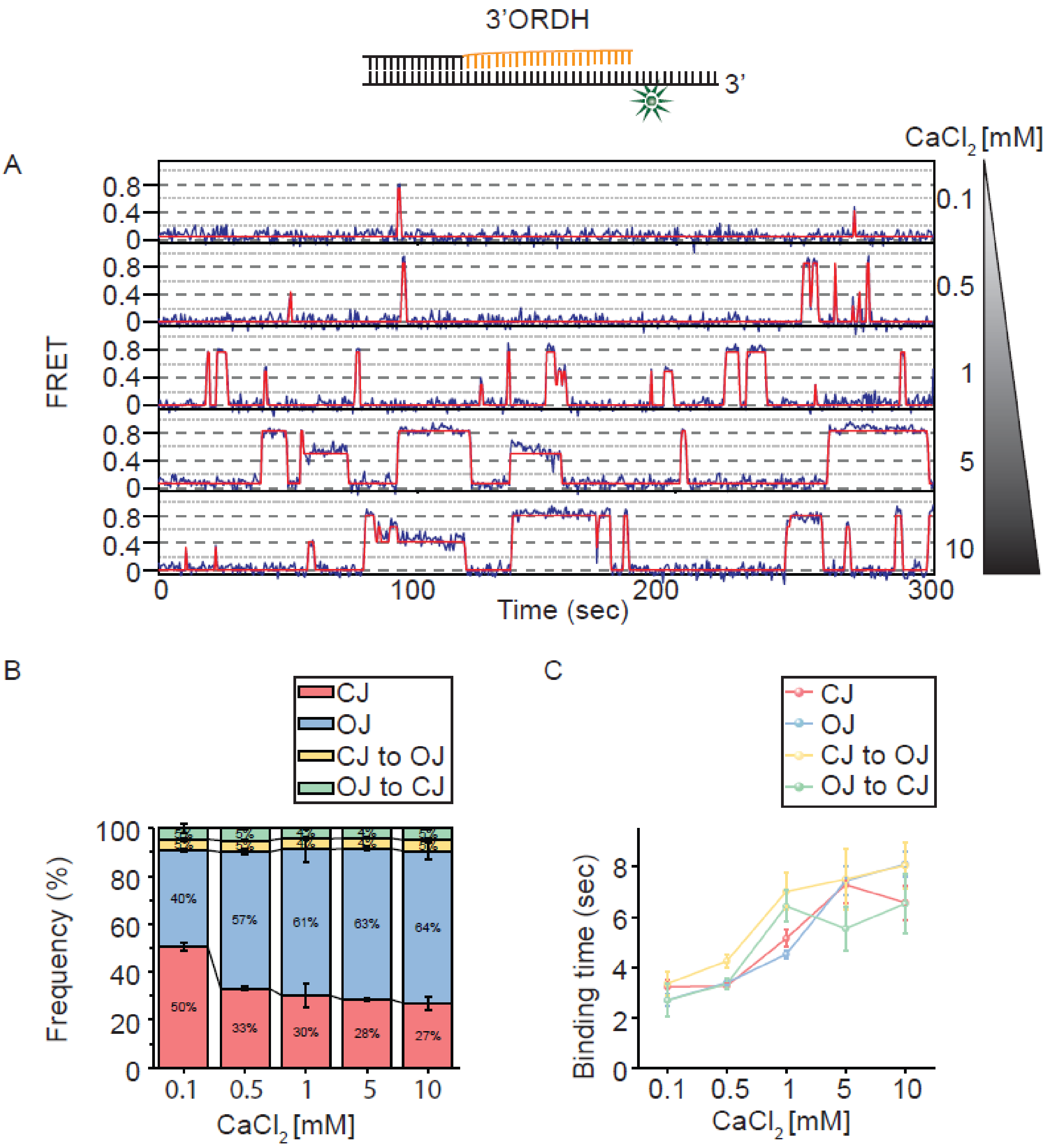

Figure S3. $CaCl_2$ serves as a binding cofactor for RNase H.

Titration of $CaCl_2$ in the range of 0.1 to 10 mM. As the $CaCl_2$ concentration increases, the binding frequency and binding time of RNase H to the substrate increase. (A) Representative FRET-time trajectories in varying concentrations of $CaCl_2$. (B) Binding frequency versus $CaCl_2$ concentration: pink (binding and dissociation at CJ), blue (binding and dissociation at OJ), yellow (diffusion from CJ to OJ), and green (diffusion from OJ to CJ). (C) Binding time versus $CaCl_2$ concentration. The binding time of RNase H to the substrate increases with increasing $CaCl_2$ concentration.

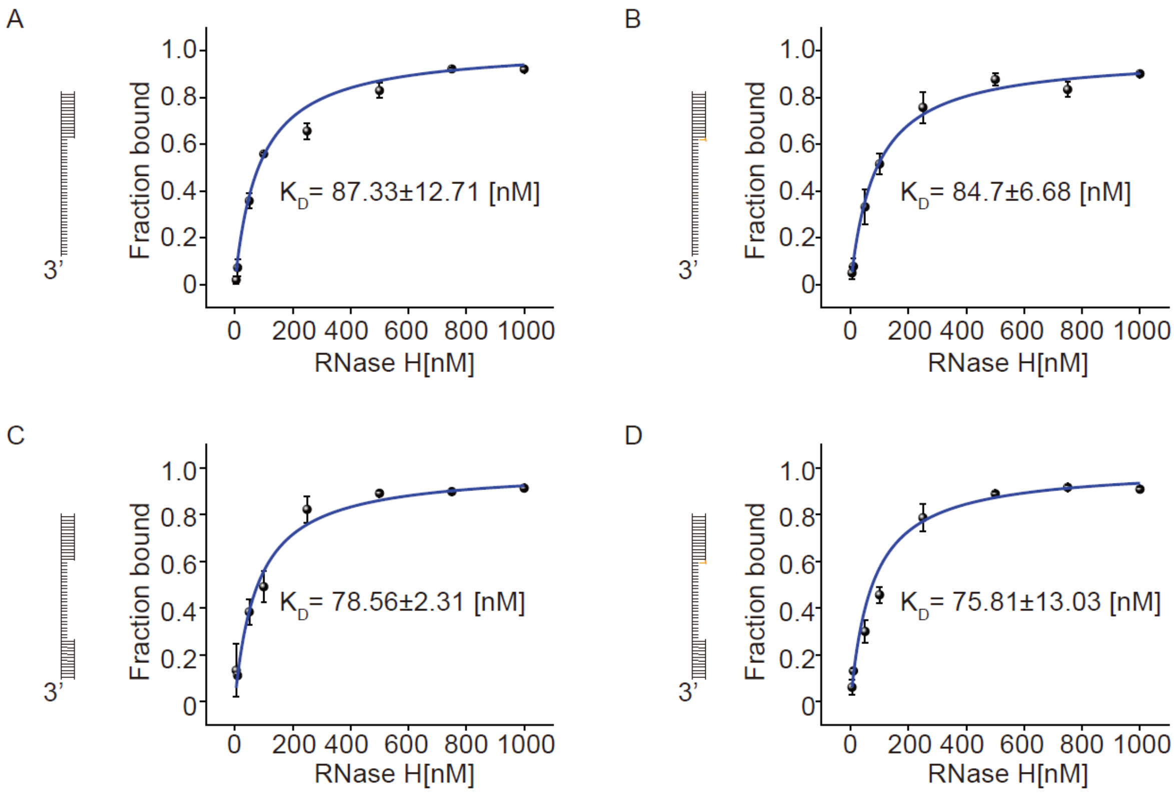

Figure S4. Binding affinity on DNA and 1 nt RNA constructs.

All binding reactions were performed with 10 pM substrate at various concentrations of RNase H in binding buffer (50 mM Tris-HCl (pH 8.3), 75 mM KCl, 10 mM $CaCl_2$, 100 µg/ml BSA, 1 mg/ml Trolox, 1 mg/ml glucose oxidase, 0.04% mg/ml catalase). The data were plotted as the fraction of bound substrates versus the concentration of RNase H (black circles, with error bars). Dissociation constants (K_d) were acquired from nonlinear fitting (blue line) using the Origin program. K_d values of different DNA constructs (A,C) without RNA and (B,D) with a 1 ribonucleotide (orange) are shown as the mean +/- s.e.m.

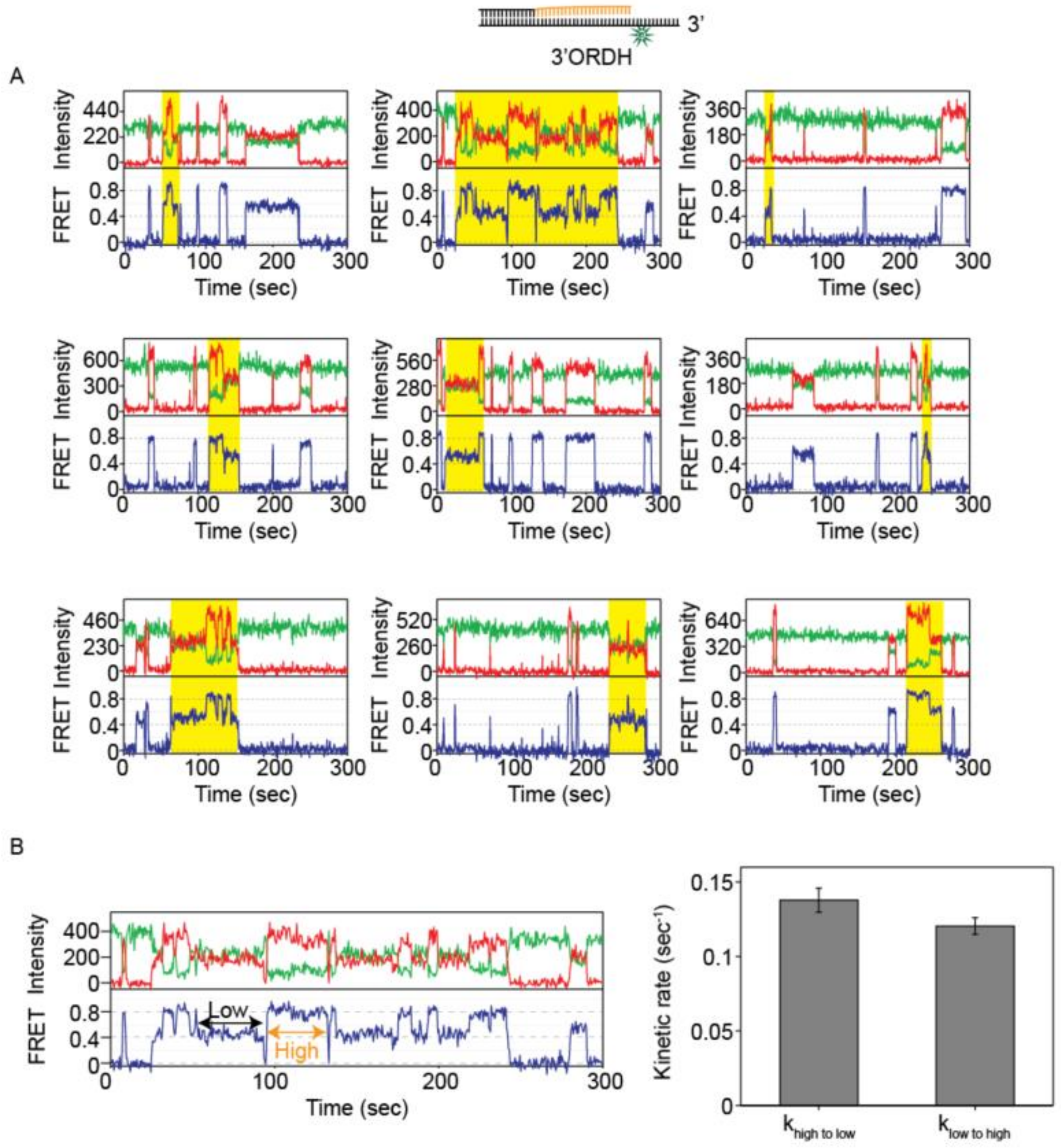

Figure S5. RNase H is capable of diffusing bilaterally between chimeric and overhang junctions along the 3’ORDH.

(A) Examples of FRET-time trajectories. ~8% of all binding events showed lateral diffusion (yellow box) scanning the 25 bp RNA:DNA heteroduplex. (B) FRET-time trace showing how $k_{(high\ to\ low)}$ and $k_{(low\ to\ high)}$ are assigned (left), and kinetic rate analysis (right). The bidirectional diffusion rates between the chimeric junction with low FRET and the overhang junction with high FRET were measured. The diffusion rates moving from high to low FRET and from low to high FRET were very similar, ~0.14±0.01 and ~0.12±0.01 sec^{-1}, respectively (B, right).

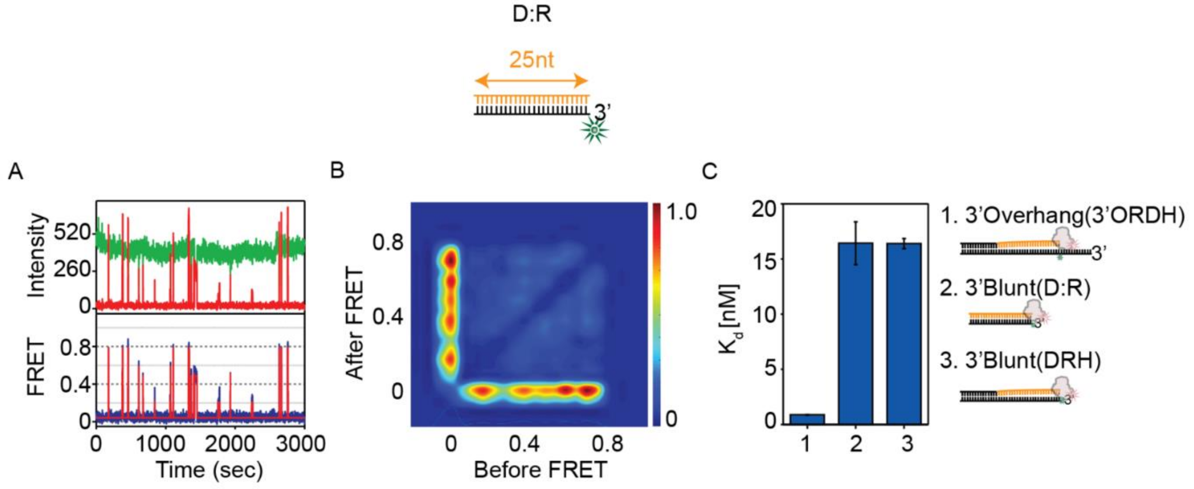

Figure S6. Assay of RNase H binding to a typical DNA:RNA (D:R).

(A) Representative FRET-time trajectory. To examine primary binding sites, Cy5-labeled RNase H was added to the Cy3-labeled 25 nt D:R substrate (top cartoon), which is mostly used in other structural and biochemical studies. (B) TDP profiled from FRET-time trajectories showed that the binding pattern of D:R did not reveal any primary binding sites, which was consistent with the fact that RNase H is a non-sequence-specific endonuclease. (C) The binding affinity of 3'OJ was ~20 times higher than that of blunt-ended hybrids, with ~0.87, ~16.43, and ~16.4 nM for 3'ORDH, D:R, and DRH, respectively (Figure 2).

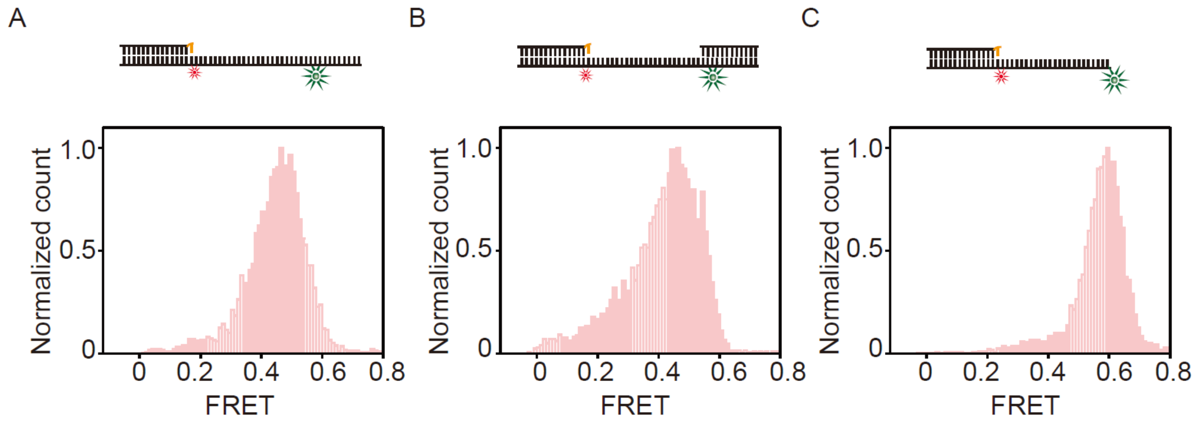

Figure S7. FRET histograms built from DNA constructs with a 1 ribonucleotide (orange) that mimics the final reaction products in the absence of RNase H. The FRET histograms were in good agreement with those after the reaction (pink) in the main Figure 4C.

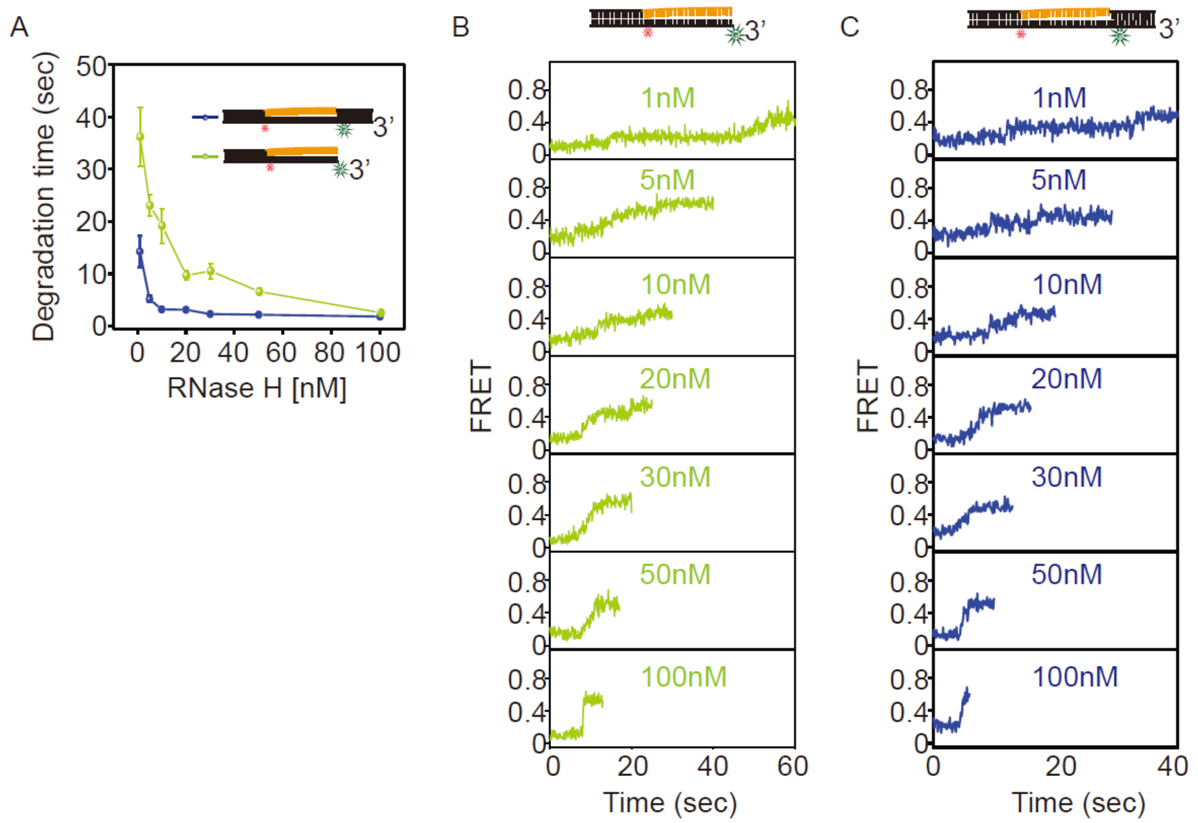

Figure S8. Distributive degradation by RNase H on RNA:DNA hybrids without a 3' overhang, performed by multiturnover reactions.

(A) Degradation time versus enzyme concentration for substrates without a 3' overhang. The degradation time of RNA nucleotides decreased with increasing concentrations of RNase H in D/RDH (blue) and RDH (green). (B) Example FRET-time trajectories from RDH at varying RNase H concentrations (1-100 nM). (C) Example FRET-time trajectories from D/RDH at varying RNase H concentrations (1-100 nM).

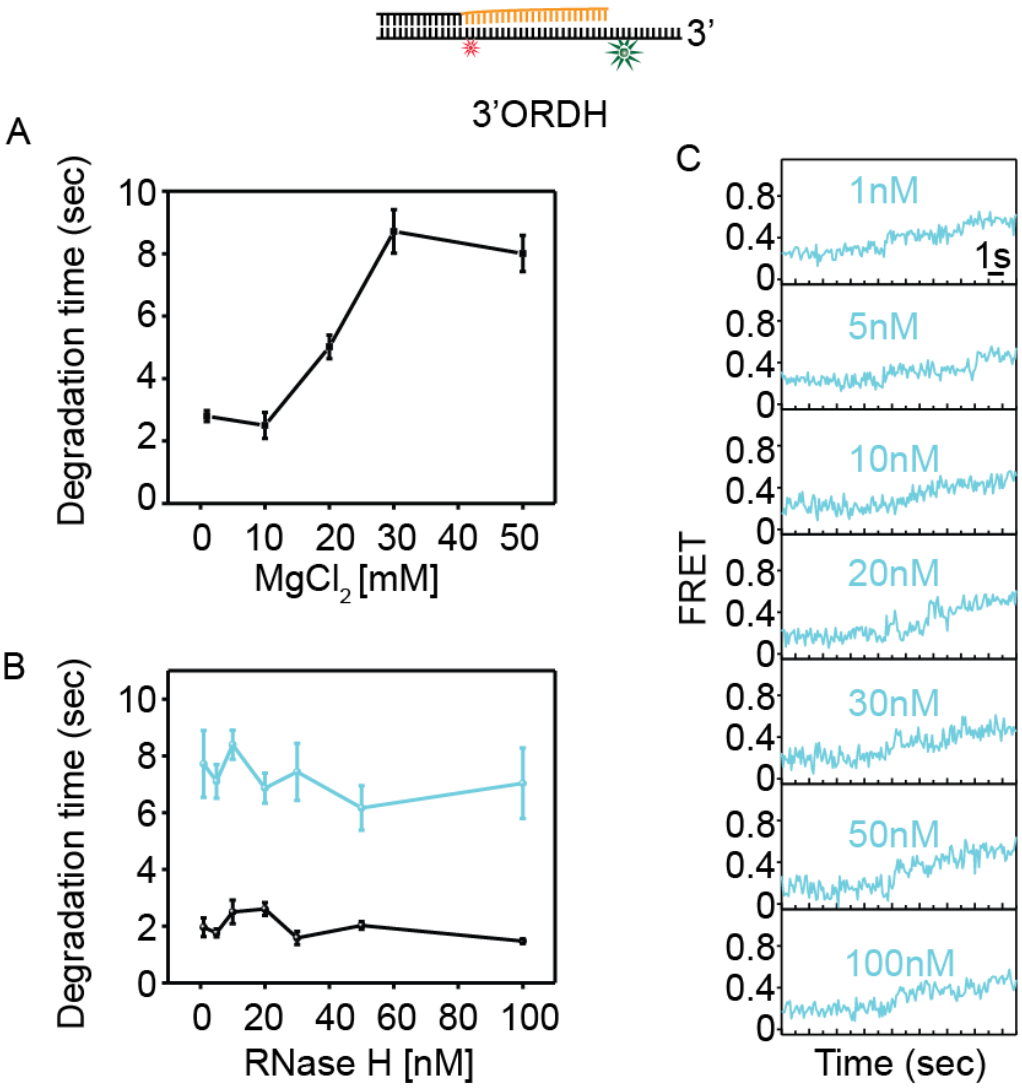

Figure S9. The protein concentration independence even at a rate 4-fold slower than the standard degradation rate of RNase H, confirming processive degradation

(A) To find a condition that significantly slowed down the degradation time, the concentration of the cofactor Mg^{2+} was varied from 1 to 50 mM. The degradation time increased ~ 4-fold at 30 mM Mg^{2+}. (B) Under this condition, the degradation time was measured by increasing the concentration of RNase H from 1 to 100 nM at Mg^{2+} concentration of 10mM (black) and 30mM (sky blue). As a result, the degradation time was constant regardless of the concentration of RNase H. (C) Representative FRET-time traces at various protein concentrations.

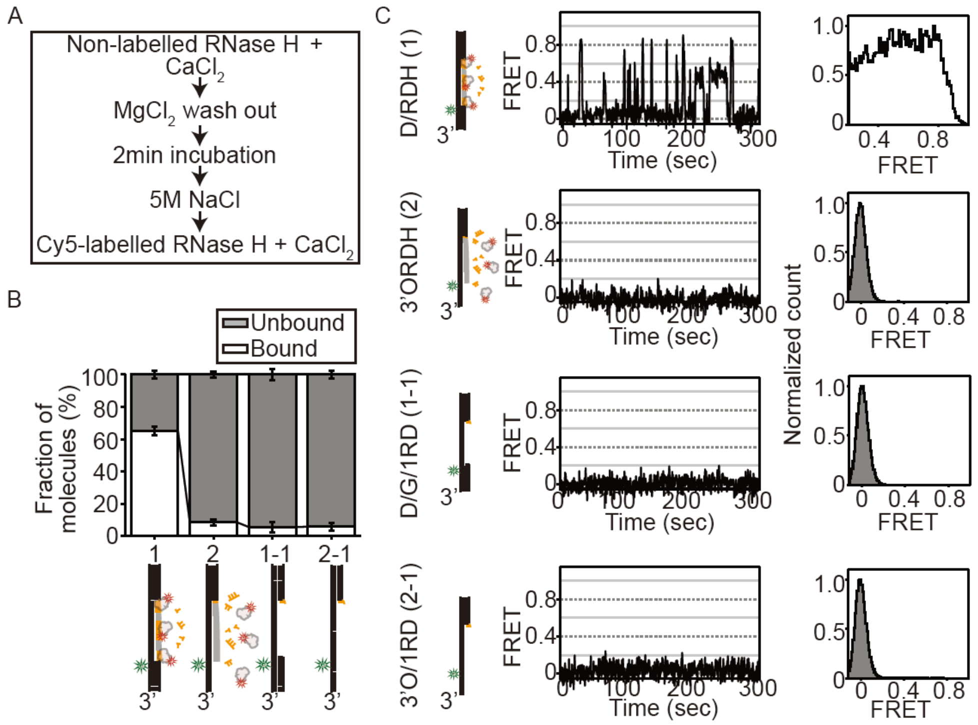

Figure S10. RNase H cannot degrade ribonucleotides on D/RDH without the 3’ overhang by single-turnover reactions due to its distributive degradation.

(A) Experimental procedures for single-turnover reactions and binding assays. Cy3-labeled substrates were immobilized on the PEGylated surface. Nonlabeled RNase H (~5 nM) was added to the hybrid substrate in the presence of Ca^{2+}, allowing RNase H to bind, and the reaction was initiated by introducing a flow of 10 mM Mg^{2+}. The flow removed free RNase H, which was not bound to the substrates; thus, only prebound RNase H performed degradation for 2 min, and 5 M NaCl was injected to quench the reaction and to remove bound RNase H from the substrates by introducing a high-salt buffer. To identify the type of degradation (i.e., processive and distributive), Cy5-labeled RNase H and Ca^{2+} were injected into the substrates with which the single-turnover reaction was previously performed. The imaging of binding events in the presence of Ca^{2+} allowed the quantification of degradation products. (B) Fractions of bound and unbound molecules profiled from various hybrid substrates, where D/RDH (1) and 3’ORDH (2) were standard substrates, and D/RDH-product (1-1) and ORDH-product (2-1) were complete degradation-mimic substrates. For complete degradation, labeled RNase H could not bind to degradation products after the single-turnover reaction, whereas for incomplete degradation, RNase H was able to bind to the products. Sixty-five percent of molecules showed binding events (1 in B and C). The other substrates ranged from 1.8% to 3.3% bound molecules (B). For D/RDH, the percentage of unbound molecules was 35%, while for the other substrates, the percentages were ~94.3% (1-2), ~91.4% (2), and ~94.1% (2-1). (C) Example FRET-time trajectories (left) and FRET distribution histogram (right) for various hybrid substrates.

In short, overall data from single-turnover reactions suggested that RNase H performs processive degradation on 3’ORDH with a 3’ ssDNA overhang.

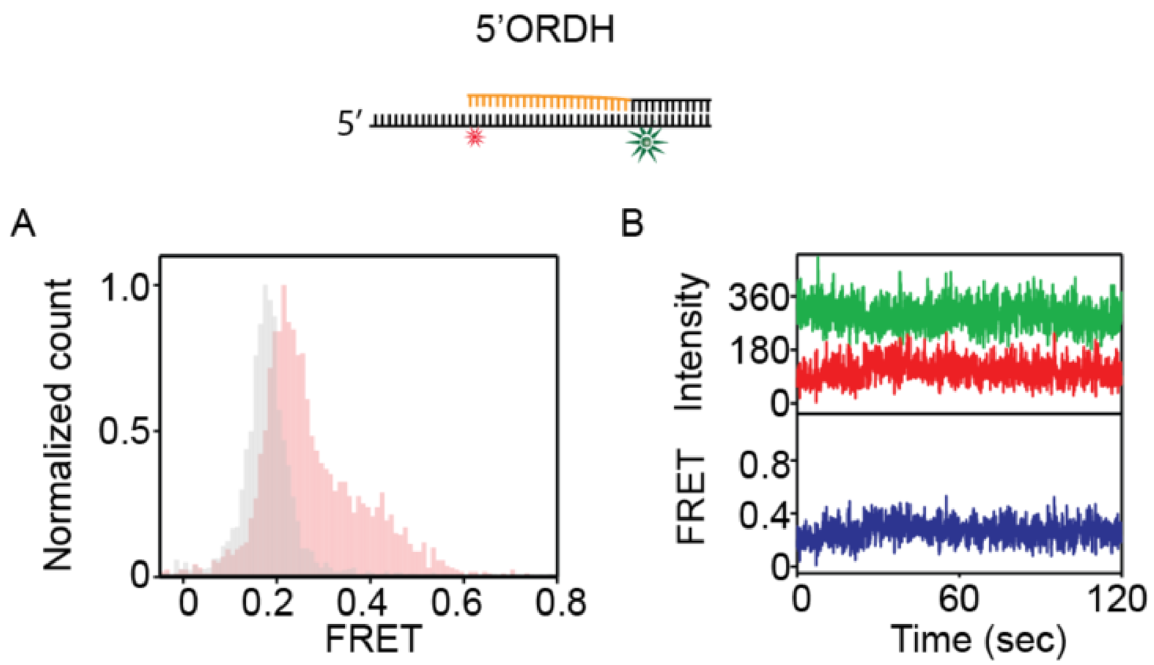

Figure S11. RNase H degrades 5’ORDH with a 5’ overhang in a distributive manner.

A single-turnover reaction was carried out on 5’ORDH with a 5’ overhang. (A) FRET histograms before (light gray) and after the reaction (pink). The FRET value after the single-turnover reaction did not reach ~0.58, which is the FRET value of complete degradation. (B) Representative FRET-time trajectory showing incomplete degradation.

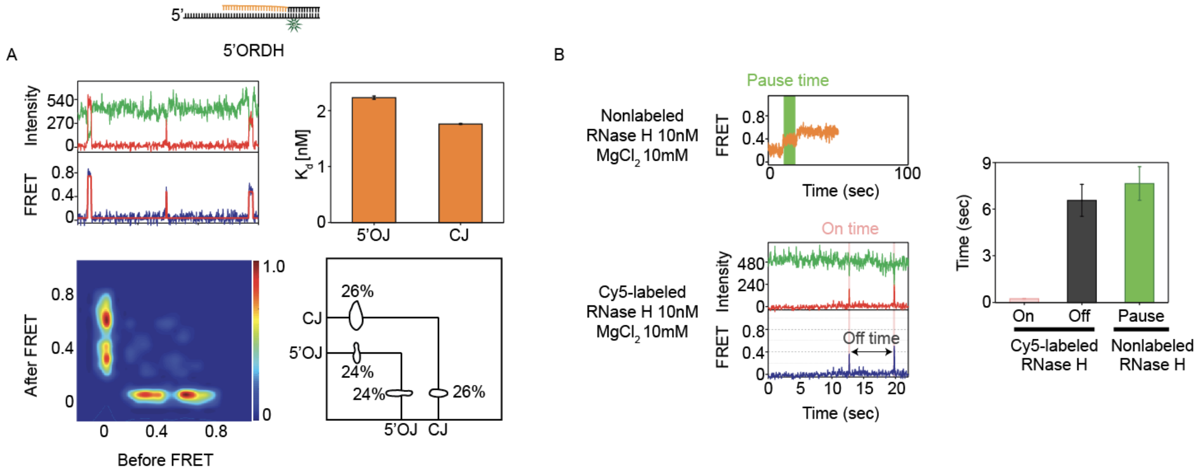

Figure S12. Binding (A) and degradation (B) assays have confirmed that RNase H performs distributive degradation activity on 5'ORDH with a 5' overhang.

(A) We performed a binding assay of RNase H on the 5'ORDH. Binding and dissociation events were observed in the same way as in the main Figures 1 and 2. Two broad population of FRET values were detected at E = 0.4 and 0.7. The low and high FRET were attributed to the binding of RNase H to the 5'OJ and CJ, and TDP revealed that binding frequencies for 5'OJ and CJ were 24% and 26%, respectively (% next to circles). Consistently, K_d of 5'OJ and CJ were ~2.23 and ~1.76 nM, respectively (bar graphs, top-right in A). This shows that binding affinities are comparable at both the junctions of 5'ORDH. (B) In order to clarify the nature of enzymatic directionality and activity on the 5'ORDH, we used Cy5-labeled RNase H to investigate whether RNase H dissociates from or stops on the substrate when FRET shows a pause. As a result, the average binding (on) and dissociation (off) times at 10 nM Cy5-lableled RNase H were ~0.23 s (pink) and ~6.56 s (black), respectively (right in B). The average pause time (top-left in Figure 12B or green arrow in Figure 6A) was ~7.65 s at 10 nM nonlabeled RNase H (green, right in B). The similarity between dissociation and pause times (i.e., ~6.56 and ~7.65 s, respectively) indicates that a pause appears in the FRET trace when RNase H dissociates from the 5'ORDH.

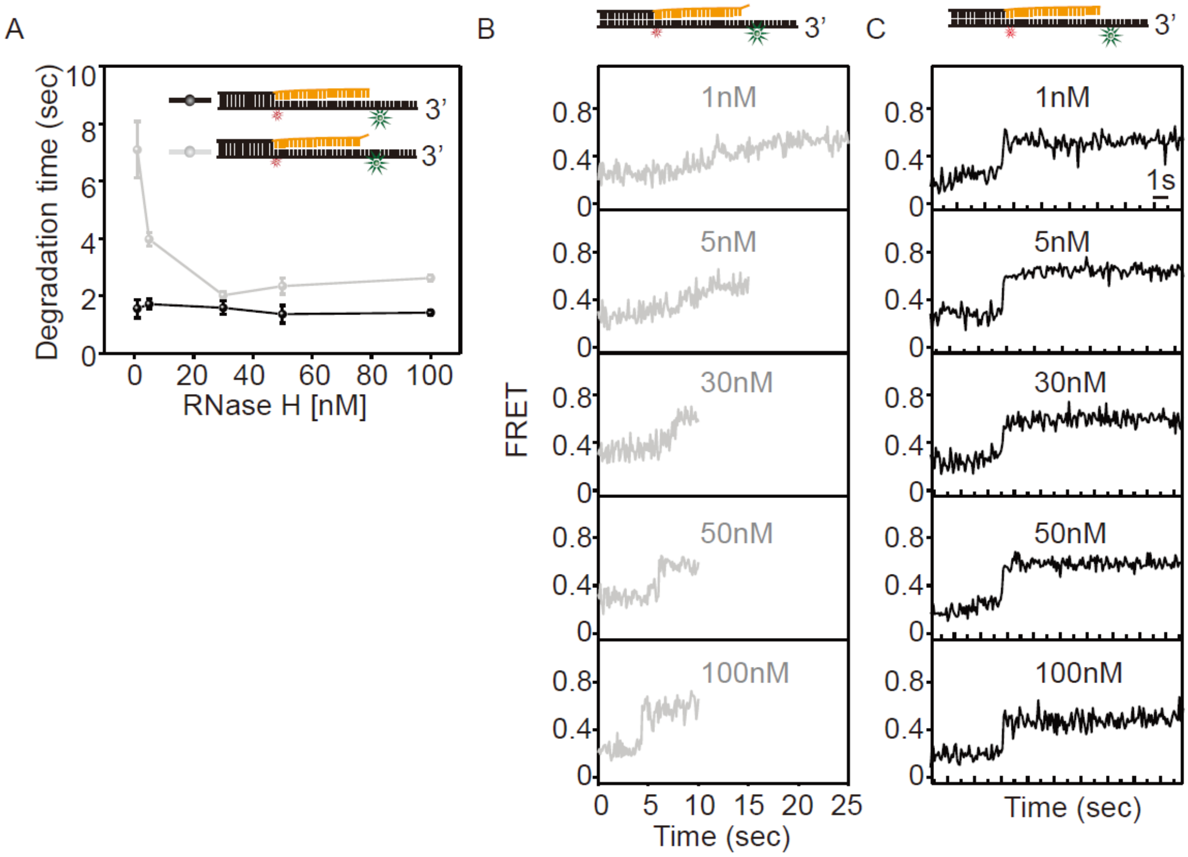

Figure S13. RNase H preferentially bound to the 3'OJ and degraded RNA.

(A) Degradation time versus enzyme concentration for 3'ORDH (black) and 3'ORDH with a 2-bp mismatch at the 5' end of the RNA at the ss-ds junction (gray). The degradation time of 3'ORDH with the 2-bp mismatch was longer than that of intact 3'ORDH. (B, C) Representative FRET-time trajectories at varying protein concentrations (1-100 nM), showing enzyme concentration-dependent and enzyme concentration-independent degradation for 3'ORDH without (C) and with the 2-bp mismatch (B).

The data also indicate that RNase H preferentially binds and degrades the RNA at the ss-ds junction (3'OJ) but not the inner RNA of 3'ORDH in the presence of a 3' ssDNA overhang. Otherwise, the mismatch effect at the 3'OJ would not appear on 3'ORDH.

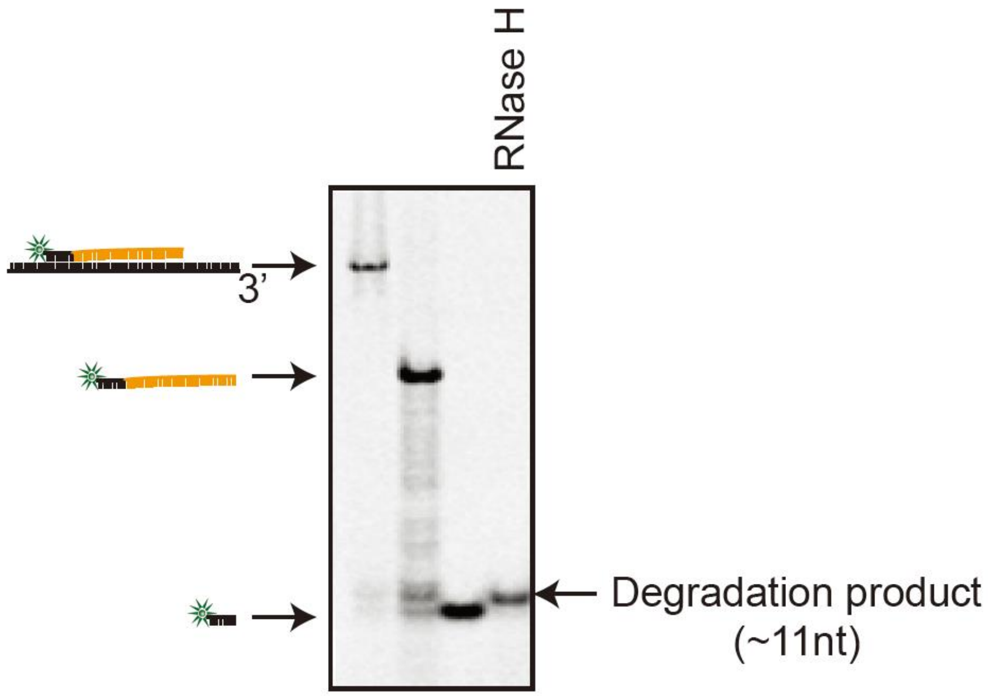

Figure S14. RNase H cannot remove the last ribonucleotide of the RNA-DNA strand (orange) at the chimeric junction. (1st-3rd lanes) ladders shown on the left. (4th lane) The degradation product of the substrate shown in the 1st lane after the 5 min reaction by RNase H at room temperature.

Table S1. Sequence information on oligos used for the study.

Construct	Sequence (5'→3')	Description
Overhang/blocked template_top	5'-/5Phos/TCT CTC CTC TCC T/iAmMC6T/C ATC ATC CTC TTC CCA CCC -3'	Top template used for ligated template (65 nt)
Overhang/blocked template_bottom	5'-/5Biosg/TTC ACA CTT CCT TCT CTC TC/iAmMC6T/TCC TCT CCC TC -3'	Bottom template used for ligated template (65,45 nt)
Blunt template top	5'-/5Phos/TCT CTC CTC TCC T/3AmMO/-3'	Top template used for ligated template (45 nt)
45 nt chimeric RNA	5'-/5Phos/rArGrG rArGrA rGrGrA rGrArG rArGrA rGrGrG rArGrA rGrGrA rAGA GAG AGA AGG AAG TGT GAA -3'	Complementary RNA (25 nt) for template
Opposite direction 45 nt chimeric RNA	5'-GGG TGG GAA GAG GAT GAT GArA rGrGrA rGrArG rGrArG rArGrA rGrArG rGrGrA rGrArG rGrArA - 3'	Complementary RNA in opposite direction for template
1 nt chimeric RNA	5'-/5Phos/rAGA GAG AGA AGG AAG TGT GAA -3'	Substrate for product form
15 nt chimeric RNA	5' -/5Phos/rArGrG rArGrA rGrGrA rGrArG rArGrA GGG AGA GGA AGA GAG AGA AGG AAG TGT GAA - 3'	Complementary RNA (15 nt) for template
10 nt chimeric RNA	5'-/5Phos/rGrGrG rArGrA rGrGrA rAGA GAG AGA AGG AAG TGT GAA-3'	Complementary RNA (10 nt) for template
2 nt mismatch RNA	5'-/5Phos/rCrArG rArGrA rGrGrA rGrArG rArGrA rGrGrG rArGrA rGrGrA rA -3'	Substrate to verify melting step
Gel assay chimeric RNA	5'-/5Phos/rArGrG rArGrA rGrGrA rGrArG rArGrA rGrGrG rArGrA rGrGrA rAGA GAG AGA AG/3AmMO/-3'	Substrate for gel assay
Chimeric DRD	5'-GGG TGG GAA GAG GAT GAT GArA rGrGrA rGrArG rGrArG rArGrA rGrArG rGrGrA rGrArG rGrArA GAG AGA GAA GGA AGT GTG AA-3'	Substrate for both-sides chimeric junction
25 nt complement RNA	5' -/5Phos/rArGrG rArGrA rGrGrA rGrArG rArGrA rGrGrG rArGrA rGrGrA rA -3'	RNA used for D/R/DH and R/DH substrates
65 nt top label template	5'-/5Biosg/TTC ACA CTT CCT TCT CTC TCT TCC TCT CCC TCT CTC TCC TCT CCT/iAmMC6T/CAT CAT CCT CTT CCC ACC C-3'	Template (65 nt) for single dye (top)
65 nt bottom label template	5'-/5Biosg/TTC ACA CTT CCT TCT CTC TC/iAmMC6T/T CCT CTC CCT CTC TCT CCT CTC CTT CAT CAT CCT CTT CCC ACC C-3'	Template for single dye (bottom)
50 nt top label template for 10 nt chimeric RNA	5'-/5Biosg/TTC ACA CTT CCT TCT CTC TCT TCC TCT CCC/iAmMC6T/CTC TCT CCT CTC CTT CAT C	Template (50 nt) for single dye (top)
10 nt DNA for gel assay control	5'-GAG AGA GAA G/3AmMO/-3'	Control substrate of gel assay
35 nt complementary top DNA	5' -GGG TGG GAA GAG GAT GAT GAT GAA GGA GAG GAG AG	Complementary DNA for control (S4)
20 nt complementary top DNA	5'-GGG TGG GAA GAG GAT GAT GA-3'	Top complementary DNA for D/RDH and D/R/DH substrate
20 nt complementary bottom DNA	5'-GA GAG AGA AGG AAG TGT GAA-3'	Bottom complementary DNA for D/R/DH substrate

65nt complementary RNA	‘5-/5Phos/UrArA rAUrA UrArA UrArA UrArA rCrC rCUrC UrCU rCUrC UrCrC rCUrC rCrCU rCUrC UrAU rCUU UUrC UUU UrCU rCUrC UrCU rCUrC UrCrC- 3’	Complementary RNA for 5’ssRNA overhang substrate
50nt complementary DNA	5’-/5Biosg/ GGA GAG AGA GAG AGA AAA GAA AAG A /iAmMC6T/ AG AGA GGG AGG GAG AGA GAG AGG G /3Cy3Sp/-3’	Complementary DNA for 5’ssRNA overhang substrate

* /5Biosg/ refers to biotinylated position for substrate immobilization

* /5Phos/ refers to phosphorylated position

* /3AmMO/ refers to amino-modified position

* /iAmMC6T/ refers to amino-modified thymine base

* /3Cy3Sp/ refers to Cy3 fluorophore-modified position

Table S2. Structures and abbreviated names of all substrates.

Abbreviation	D/RDH	D/R/DH	3’ORDH (25R)	3’OR/DH
Substrate	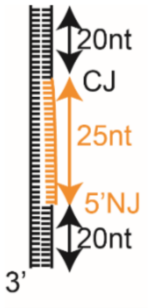	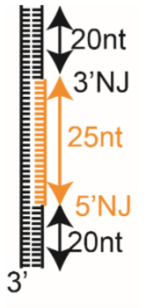	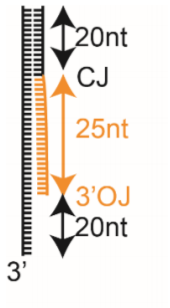	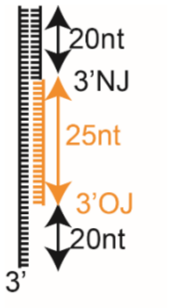
Abbreviation	RDH	R/DH	3’OR5’OH	D:R
Substrate	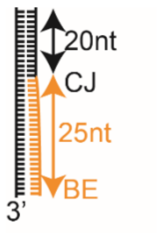	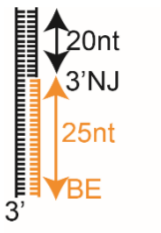	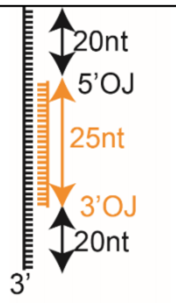	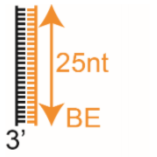
Abbreviation	5’ORDH	3’ORDH (15R)	3’ORDH (10R)	D/G/1RD
Substrate	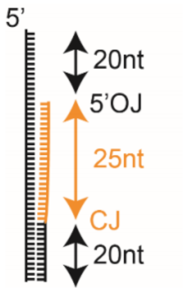	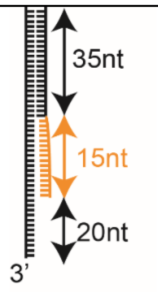	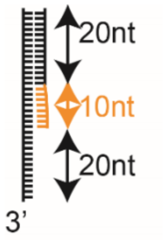	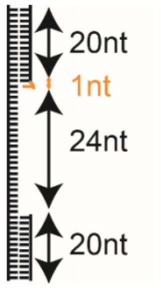
Abbreviation	D/G/D	3’O/1RD	3’O/D	5’RORDH
Substrate	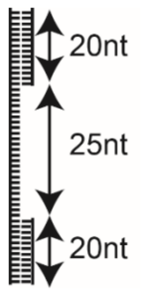	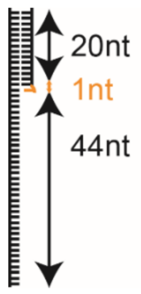	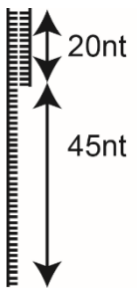	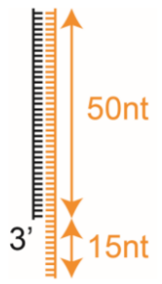

Table S3. Statistics of all Figures.

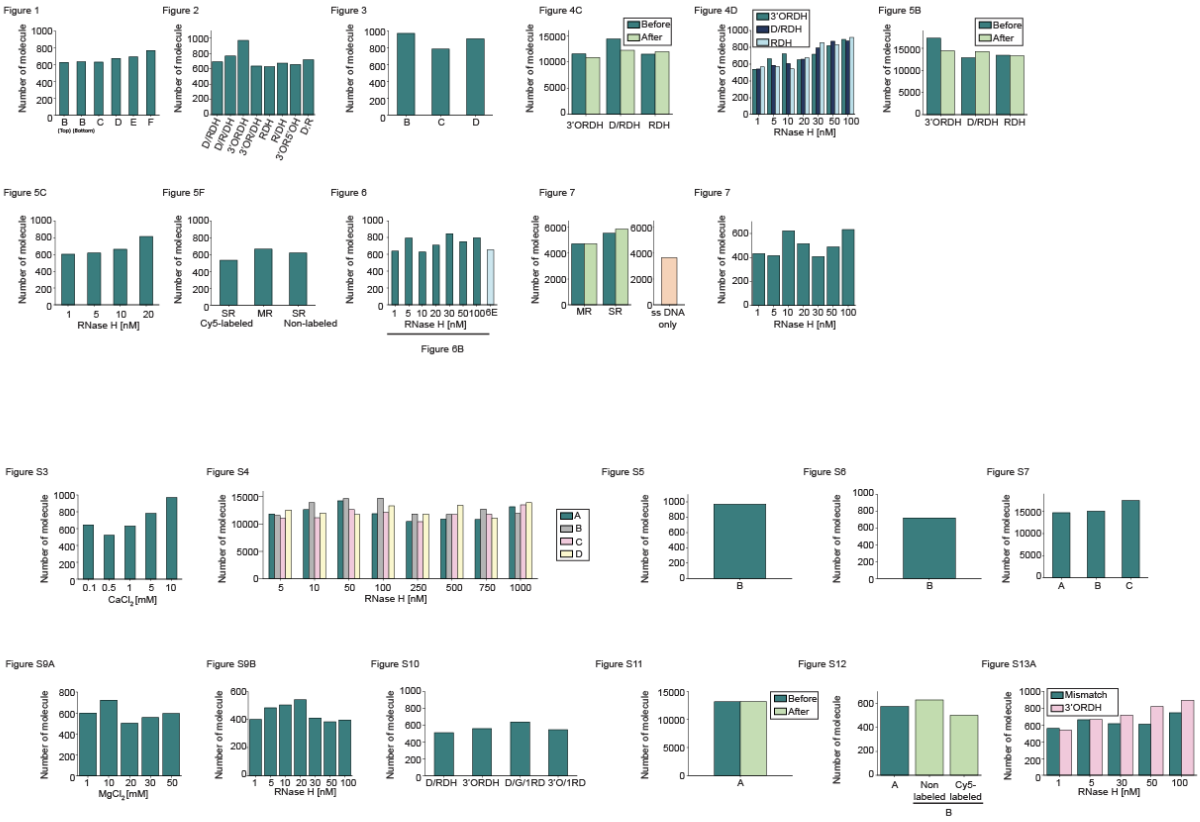

Supplement: gkab1064_Supplemental_File [file gkab1064_supplemental_file.pdf]
